# Comprehensive analysis of sesame LRR-RLKs: structure, evolution and dynamic expression profiles under *Macrophomina phaseolina* stress

**DOI:** 10.3389/fpls.2024.1334189

**Published:** 2024-02-12

**Authors:** Wenqing Yan, Yunxia Ni, Hui Zhao, Xintao Liu, Min Jia, Xinbei Zhao, Yongdong Li, Hongmei Miao, Hongyan Liu, Haiyang Zhang

**Affiliations:** ^1^ The Shennong Laboratory, Zhengzhou, Henan, China; ^2^ Institute of Plant Protection, Henan Academy of Agricultural Sciences, Key Laboratory of IPM of Pests on Crop (Southern North China), Ministry of Agriculture, Key Laboratory of Crop Pest Control of Henan, Zhengzhou, Henan, China; ^3^ Key Laboratory of Specific Oilseed Crops Genomics of Henan Province, Henan Sesame Research Center, Henan Academy of Agricultural Sciences, Zhengzhou, Henan, China

**Keywords:** *Sesamum indicum*, LRR-RLK, evolution, expression profiles, *Macrophomina phaseolina*

## Abstract

Leucine-rich repeat receptor-like kinases (LRR-RLKs) can participate in the regulation of plant growth and development, immunity and signal transduction. *Sesamum indicum*, one of the most important oil crops, has a significant role in promoting human health. In this study, 175 *SiLRR-RLK* genes were identified in *S. indicum*, and they were subdivided into 12 subfamilies by phylogenetic analysis. Gene duplication analysis showed that the expansion of the *SiLRR-RLK* family members in the sesame was mainly due to segmental duplication. Moreover, the gene expansion of subfamilies IV and III contributed to the perception of stimuli under *M. phaseolina* stress in the sesame. The collinearity analysis with other plant species revealed that the duplication of *SiLRR-RLK* genes occurred after the differentiation of dicotyledons and monocotyledons. The expression profile analysis and functional annotation of *SiLRR-RLK* genes indicated that they play a vital role in biotic stress. Furthermore, the protein−protein interaction and coexpression networks suggested that SiLRR-RLKs contributed to sesame resistance to *Macrophomina phaseolina* by acting alone or as a polymer with other SiLRR-RLKs. In conclusion, the comprehensive analysis of the *SiLRR-RLK* gene family provided a framework for further functional studies on *SiLRR-RLK* genes.

## 1 Introduction

Receptor-like protein kinases (RLKs) represent numerous transmembrane kinases that sense stimulation at the cell surface and mediate cell signal transduction by phosphorylation in response to the environment (Trenker and Jura, 2020). Many duplication events of RLKs exist in terrestrial plants (Lehti-Shiu et al., 2009), in which RLKs involved in the stress response show duplications, while those involved in growth and development do not (Shiu et al., 2004), suggesting that duplication events of RLKs are important for terrestrial plants to respond to ever-changing environments (Lehti-Shiu et al., 2012). LRR-RLKs represent the largest family in RLKs, which consist of three protein domains: an LRR domain sensing signal outside the cell, a single-channel transmembrane domain anchoring proteins within the membrane, and a kinase domain involved in signal transduction by autophosphorylation and subsequent specific substrate phosphorylation (Liu et al., 2017).

LRR-RLKs can widely regulate plant development and stress responses by participating in brassinosteroid (BR) and abscisic acid (ABA) signaling pathways. BRI1 (BR insensitive 1), a key LRR-RLK in the BR pathway, could regulate stem elongation, vascular differentiation, seed size, fertility, flowering time and senescence by BR signaling in *Arabidopsis* by forming the BRI1/BAK1 (BRI1-associated receptor kinase 1) complex (Li et al., 2002; Nam and Li, 2002; Wang et al., 2005). In addition, barley *bri1* mutant have multiple effects on disease resistance and plant developmental regulation (Goddard et al., 2014). SERK2 (Somatic embryogenesis receptor kinase 2), another component of the BR pathway, can mediate salt tolerance in rice via BR signaling (Dong et al., 2020). Moreover, OsSERK2 confers rice immunity to *Xanthomonas oryzae* pv. *oryzae* by activating the resistance genes *XA21* and *XA3* (Chen et al., 2014). BAK1 plays an important role in ABA signaling in guard cells. The *bak1* mutants exhibited more water loss than the wild type and showed ABA insensitivity in stomatal closure. Additionally, ABA can facilitate the formation of the BAK1/OST1 (Open stomatal 1) complex that mediates ABA-induced stomatal closure in guard cells near the plasma membrane (Shang et al., 2016). Likewise, KIN7 (Kinase 7) is essential in ABA signaling in stomatal closure. Phosphorylation and activation of TPK1 (Tonoplast K^+^ channel) by the KIN7 is indispensable for ABA- and CO_2_-mediated stomatal closure (Isner et al., 2018). In addition, LRR-RLKs have been shown to be involved in plant immunity via other phytohormone pathways. PSKR1 (Phytosulfokine receptor 1), an antagonistic regulator between biotrophic and necrotrophic pathogens in plant defense, can mediate plant resistance to pathogens by suppressing salicylic acid-dependent defense while enhancing jasmonic acid-dependent defense (Mosher et al., 2013). However, OsPSKR1 is involved in rice resistance to *Pseudomonas syringae* DC3000 in rice by activating the expression of *PR* genes involved in the salicylic acid signaling pathway (Yang et al., 2019). Furthermore, PEPR1 (Pep1 receptor 1) and PEPR2 are involved in plant immunity due to ROS (Reactive oxygen species) production and ethylene signaling (Ma et al., 2016).

The interaction and regulation between LRR-RLK members is intricate during development and stress. For instance, the CLV1 (CLAVATA 1)-CLV2-CRN (CORYNE) trimer is essential in plant stem cell regulation (Bleckmann et al., 2010; Zhu et al., 2010), and the BAK1-TMM (TOO MANY MOUTHS) complex is involved in plant immunity (Jordá et al., 2016). The formation of some LRR-RLK complexes depends on ligand stimulation, for instance, flg22 and elf18 can stimulate FLS2 (Flagellin sensitive 2) and EFR (Elongation factor-Tu receptor) to form dimers with BAK1 and then plant defense is initiated (Roux et al., 2011). Similarly, ligands SCFE1 (Sclerotium culture filtrate ELICITOR1) or NLP20 (Peptide motif) stimulate the formation of the BAK1-SOBIR1 (Suppressor of BIR1-1)-RLP23 complex (Gao et al., 2009), which plays an important role in plant resistance to pathogens. Moreover, there have been fewer studies on the interactions of LRR-RLKs in other plants, with only a few having been confirmed in tomato (Peng and Kaloshian, 2014), tobacco (Franco-Orozco et al., 2017), rice (Chen et al., 2014), *Medicago truncatula* (Crook et al., 2016) and wheat (Singh et al., 2016).

The large number, the great diversity of structure and function and the intricate interaction networks of *LRR-RLKs* present a challenge in understanding the functions and mechanisms of *LRR-RLK* genes in complex signal transduction pathways in plants. Furthermore, the complementary functions between LRR-RLKs indicate the importance of systematic analysis using bioinformatics tools to understand the roles of LRR-RLKs in plants. Recently, the *LRR-RLK* gene family has been reported in *Arabidopsis* (Shiu and Bleecker, 2001), soybean (Zhou et al., 2016), wheat (Shumayla et al., 2016), cotton (Sun et al., 2018), rice (Sun and Wang, 2011) and maize (Song et al., 2015). Additionally, the potential roles of LRR-RLKs in response to stresses have been well-studied in *Thinopyrum elongatum* (Mishra et al., 2021). Sesame charcoal rot caused by *M. phaseolina* is one of the most serious fungal diseases in sesame production, and threatens the yield and quality of sesame. Although *LRR-RLKs* are crucial in plant immunity, there is still a lack of systematic studies of the *LRR-RLKs* in sesame. It is of great practical significance to study *LRR-RLK* gene family in sesame and their functions related to biotic stresses. In this study, the *LRR-RLK* gene family in sesame was comprehensively analyzed by phylogeny, structural evolution and expression profile analysis. The potential functions of the sesame LRR-RLK homologous to *Arabidopsis* were predicted by protein−protein interaction (PPI) and coexpression networks. Our studies tend to gain insight into the functions of the sesame *LRR-RLK* family and provide new insights into their roles in regulation under *M. phaseolina* stress at the transcriptome level.

## 2 Materials and methods

### 2.1 LRR-RLK gene discovery in *S. indicum*

Sesame genome and proteome sequences were provided by Henan Sesame Research Center, Henan Academy of Agricultural Sciences (Zhang et al., 2013; Miao et al., 2023). HMM (Hidden Markov Model) profiles of LRRs (PF00560, PF07723, PF07725, PF12799, PF13306, PF13516, PF13855, PF14580 and PF01816) and Pkinase/Pkinase_Tyr (PF00069 and PF07714) were used for identification of putative *LRR-RLKs* in *S. indicum* through HMMER 3.1 (Finn et al., 2011) (E-value < 1 × 10^−10^). LRR-RLKs identified from *Arabidopsis* (Shiu and Bleecker, 2001), soybean (Zhou et al., 2016), wheat (Shumayla et al., 2016), cotton (Sun et al., 2018), rice (Sun and Wang, 2011) and maize (Song et al., 2015) were used to run BLASTP with the sesame proteome (E-value < 1 × 10^−5^). The sum putative LRR-RLKs of the HMMER search result and BLASTP result were used for subsequent analysis. The InterPro database (https://www.ebi.ac.uk/interpro/) was used to confirm the presence of the kinase domain, LRR domain and transmembrane domain in LRR-RLKs in sesame. Sequences that met the above conditions were regarded as *LRR-RLKs*.

### 2.2 Phylogenetic, structural and functional analysis of LRR-RLKs

The theoretical isoelectric points (*pI*) and molecular weights (MW) of SiLRR-RLKs were predicted with Expasy (https://www.expasy.org/). CELLO (http://cello.life.nctu.edu.tw/) was used to predict the subcellular localization while SignalP-5.0 (https://services.healthtech.dtu.dk/service.php?SignalP-5.0) was used for signal peptide prediction. A conserved domain analysis of the sesame SiLRR-RLK family members was performed using the InterPro database. The MEME online server (http://meme-suite.org/) was used to search for conserved motifs. The conserved domains and gene structure of SiLRR-RLKs were visualized by TBtools (Chen et al., 2020). GO annotation was performed on PANNZER 2 (http://ekhidna2.biocenter.helsinki.fi/sanspanz/).

A multiple sequence alignment was performed using ClustalW with the default parameters method based on the aa sequences of the SiLRR-RLK proteins. MEGA 7 software (Kumar et al., 2016) was used to construct a phylogenetic tree of LRR-RLK using the neighbor Joining (NJ) method, and the bootstrap value was set to 1,000. Then the phylogenetic tree was visualized and edited on the iTOL website (https://itol.embl.de/).

### 2.3 Chromosomal arrangement and gene duplication of LRR-RLK genes

The sesame genome file (In fna format) and the genome annotation file (In gff3 format) were used to visualize the chromosome localization with TBtools (Chen et al., 2020). The MCScanX (Wang et al., 2012) program was used to determine collinear orthologous gene duplications (Tandem and segmental duplications) among the sesame *LRR-RLK* gene family and syntenic *LRR-RLK* genes between sesame and other plant species. The genome files and annotation files of *Solanum tuberosum*, *Glycine max*, *Solanum lycopersicum*, *M. truncatula*, *A. thaliana*, *Vitis vinifera*, *Gossypium hirsutum*, *Hordeum vulgare*, *Zea mays*, *Triticum aestivum*, *Oryza sativa*, *Musa acuminata*, *Setaria italica* and *Sorghum bicolor* were downloaded from the Phytozome database (Goodstein et al., 2012).

### 2.4 *In silico* and *in vitro* expression analysis of LRR-RLK genes

RNA-seq data PRJNA892254 was used for *in silico* expression analysis of diverse sesame tissues. Flower tissues of variety *S. indicum* var. ‘Zhengzhi No.13’ that showed consistent growth were sampled, and the locations were marked. The capsules at the markers were sampled along with all other tissues (Roots, stems, leaves, capsules and seeds) two weeks later. For *in silico* expression analysis of sesame seed development, RNA-seq data of variety *S. indicum* var. ‘Wanzhi No.2’ during seed development comprising 7 days after flowering (7 DAF, S1), 14 DAF (S2), 21 DAF (S3) and full maturity (28 DAF, S4) were used (PRJNA739094) (Zhang et al., 2021). For *in silico* expression analysis, the RNA-seq data of the disease-resistant variety *S. indicum* var. ‘Zhengzhi No.13’ infected with *M. phaseolina* and root tissues were concomitantly collected at 0 h, 12 h, 24 h, 36 h and 48 h post inoculation (PRJNA706471). The data above were downloaded from the SRA database. The reads were filtered, and trimmed using fastp (Chen et al., 2018), then clean reads were mapped to the sesame genome with HISAT2 (version:2.0.4) (Pertea et al., 2015; Pertea et al., 2016). Finally, the FPKM value of each gene was calculated by trimmed mean of M values method (Robinson and Oshlack, 2010).

For *in vitro* expression analysis of sesame leaves under phytohormone treatment, variety *S. indicum* var. ‘Zhengzhi No.13’ sesame plants were treated by spraying with 1 mM ABA, SA and MeJA when they grew to 4 pairs of true leaves period. Leaf tissues before treatment and treated post 1h, 3h, 6h, 12h, 24h, 36h and 48h were taken for RNA extraction, reverse transcription and qPCR. The primers of selected 6 *SiLRR-RLK* genes are listed in **Supplementary Table S1**, UBQ5 gene was used as a reference gene. There were three replicates for each treatment. The samples were stored at -80°C.

### 2.5 Protein−protein interaction network of LRR-RLK proteins

The STRING database (https://string-db.org/) was used to analyze the interaction of sesame LRR-RLK proteins based on orthologs in *Arabidopsis* with a confidence parameter set at a 0.85 threshold.

## 3 Results

### 3.1 Phylogenetic analysis and physicochemical attributes of SiLRR-RLKs

**Figure 1:**
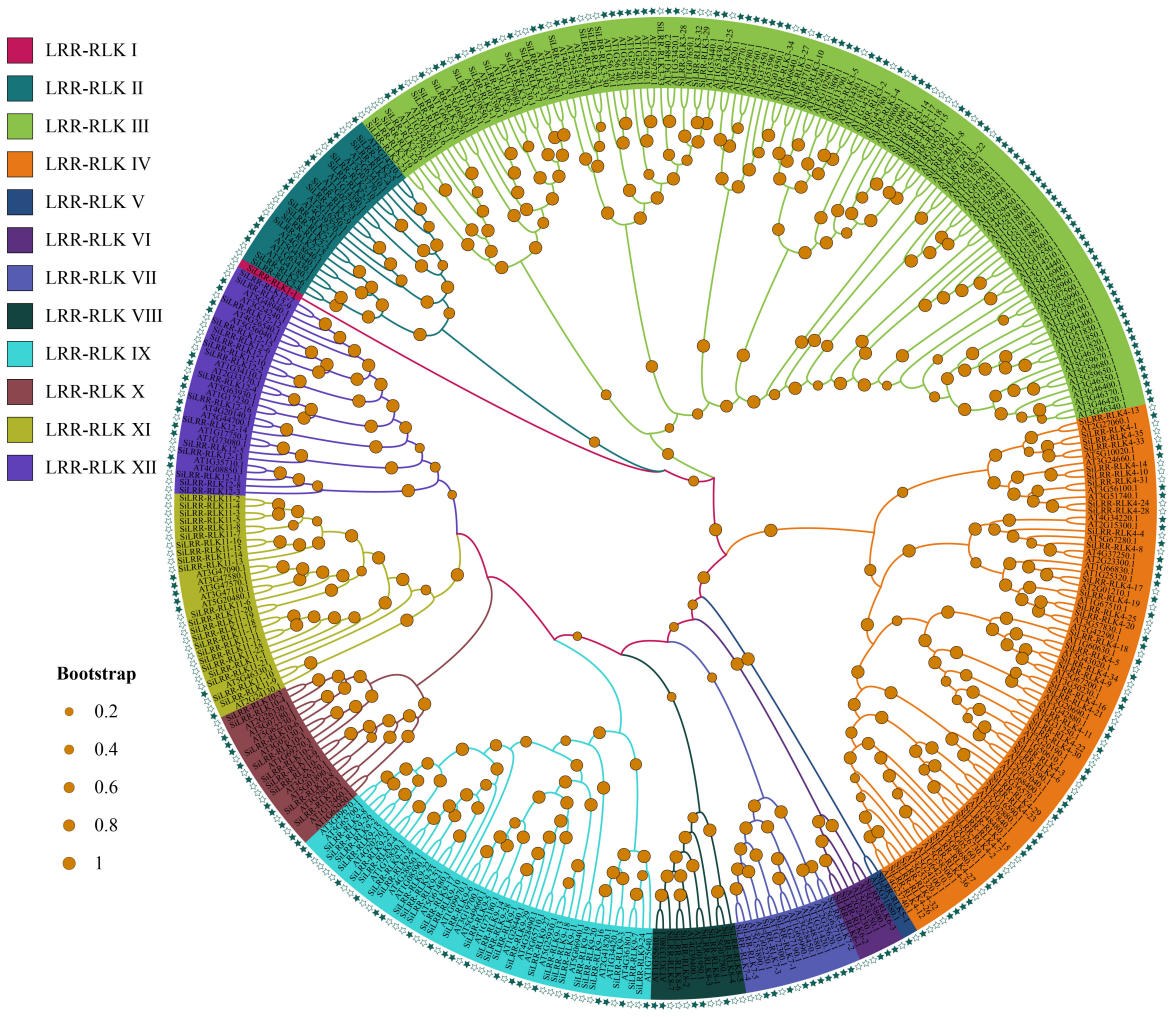
Phylogenetic analysis of the LRR-RLK proteins in *S. indicum* and *A. thaliana*. Green hollow pentacles represent LRR-RLKs in *S. indicum* while green solid pentacles represent those in *A. thaliana*.

Based on a comprehensive search of *LRR-RLK* genes by HMM profiles and BlastP, 175 LRR-RLK proteins were identified in the sesame genome. The identified *LRR-RLK* members were given names with the prefix ‘Si’ indicating *S. indicum*. Phylogenetic analysis of LRR-RLK protein sequences in *S. indicum* and *A. thaliana* was carried out (**Figure 1**). The LRR-RLK of *S. indicum* was divided into 12 subfamilies together with those of *A. thaliana*. Group IV had the most members (36), followed by 34 members in group III and 29 members in group IX (**Table 1**). These three groups comprised 56.57% of SiLRR-RLKs (**Figure 1**). Group I and V comprising only one member were the smallest subfamily (**Table 1**). The details about the SiLRR-RLK family, including their accession numbers and characteristics, were given in **Supplementary Table S2**.

**Table 1:** Subfamily designation and physico-chemical properties of the identified SiLRR-RLKs.

Subfamily	Protein Number	Aa Length	MW (kDa)	pI	Aliphatic Index
LRR-RLK I	1	1107	123.73	5.66	90.97
LRR-RLK II	12	646-908	70.9-98.99	5.38-8.75	93.07-105.15
LRR-RLK III	34	579-1042	64.21-115.06	5.53-8.88	84.06-100.3
LRR-RLK IV	36	466-1107	50.38-119.62	5.56-9.42	90.04-104.32
LRR-RLK V	1	639	71.35478	9.09	97.81
LRR-RLK VI	3	687-855	76.65-94.15	5.7-9.56	90.54-96.25
LRR-RLK VII	5	605-1075	67.11-118.71	5.61-6.81	95.85-102.94
LRR-RLK VIII	7	1099-1304	120.92-141.43	5.14-6.16	96.87-109.68
LRR-RLK IX	29	619-1145	68.77-123.81	5.18- 8.8	96.24-108.76
LRR-RLK X	9	972-1135	104.44-122.7	5.47-8.92	99.44-106.51
LRR-RLK XI	22	522-1224	57.15-134.1	5.36-7.49	103.94-111.44
LRR-RLK XII	16	932-1222	102.53-134.7	5.18-6.97	102.63-112.15

The physicochemical properties of SiLRR-RLKs enabled us to gain insight on their functions. The amino acid (aa) length of SiLRR-RLKs ranged from 466 to 1304 aa. Their isoelectric points (*pI*) were between 5.14 and 9.56, and their molecular weights ranged from 50.38 to 141.43 kDa (**Supplementary Table S2**). The summarized information regarding each subfamily was listed in **Table 1**.

### 3.2 Gene compositions, protein structure and functional annotation of SiLRR-RLKs

**Figure 2:**
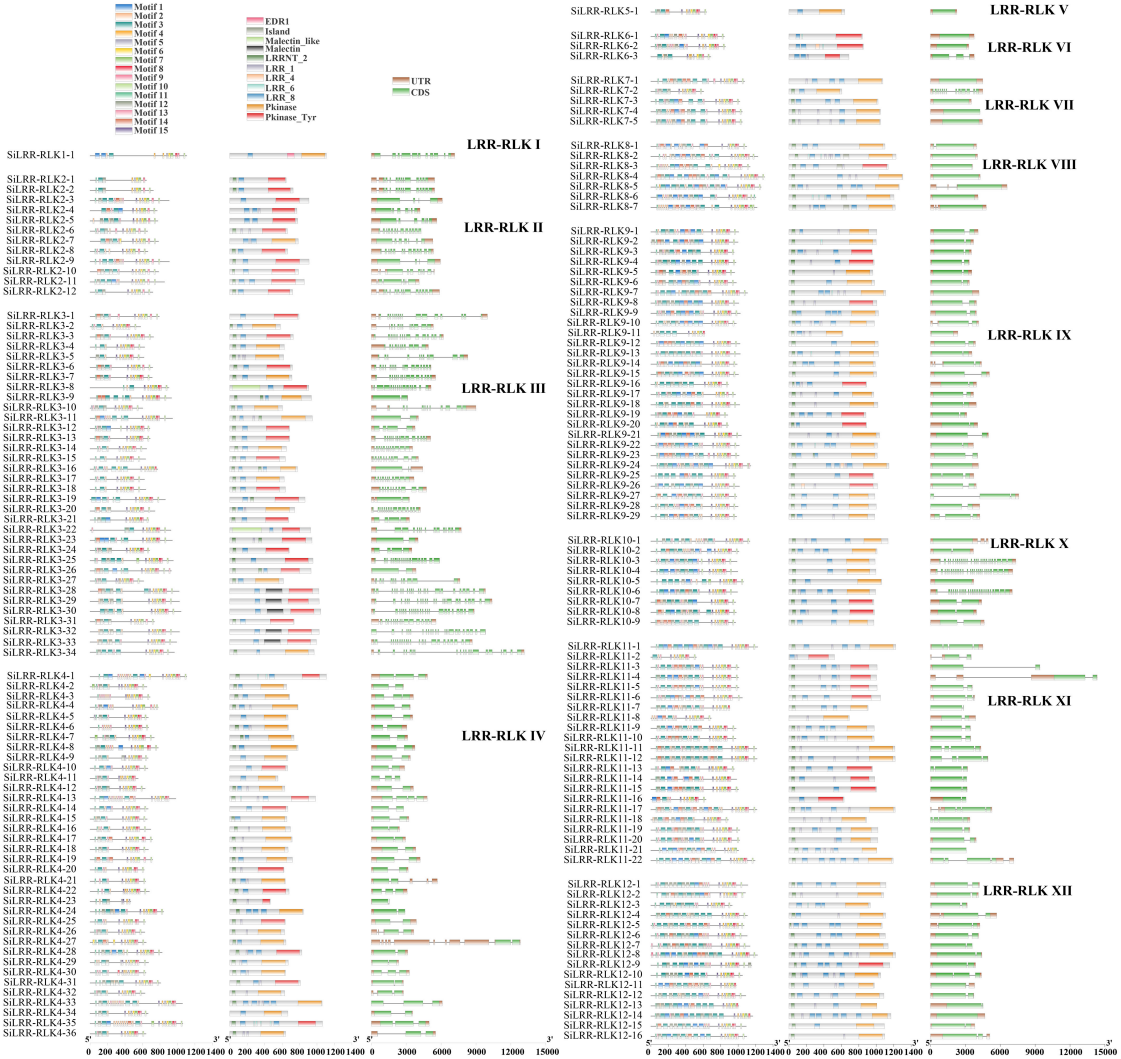
Conserved motifs, conserved domains and gene structures of 12 LRR-RLK subfamilies in *S. indicum*.

The conserved domains of proteins are closely related to their functions. Based on subfamily classification, conserved motif of *SiLRR-RLKs* were performed. Results showed that arrangement of motifs in same subfamily were similar (**Figure 2**). In addition, we identified the conserved domains of SiLRR-RLK proteins and found that they all contain both LRR and kinase domains (**Figure 2**), illustrating the accuracy of the *SiLRR-RLK* gene family. LRR-RLKs play a vital role in perceiving signals. Accordingly, a total of 78.29% of SiLRR-RLKs comprised signal peptides in our study (**Supplementary Table S2**). LRRNT_2 (PF08263) and LRR_8 (PF13855) constituted the major recognition domains in SiLRR-RLKs, which were found in 86.29% and 87.43% of SiLRR-RLKs, respectively. Furthermore, the brassinosteroid receptor island (PF20141) and Malectin (PF11721) domains were found in subfamilies III and VIII, implicating their additional roles in the recognition of BR and other plant hormones. Analysis of conversed motif and domain showed that kinase domains in LRR-RLKs C-terminal were more conserved, illustrating their potential roles in signal transduction. The structural compositions of the *SiLRR-RLK* genes were also further analyzed (**Figure 2**). The majority of *SiLRR-RLK* genes were composed of multiple exons, while only 16 *SiLRR-RLKs* were intron−less. *SiLRR-RLK10-3*, *SiLRR-RLK10-4* and *SiLRR-RLK10-6* have the most 27 exons. The exon−intron arrangement of *LRR-RLK* genes was conserved in same subfamilies while it varied in subfamilies III and X.

**Figure 3:**
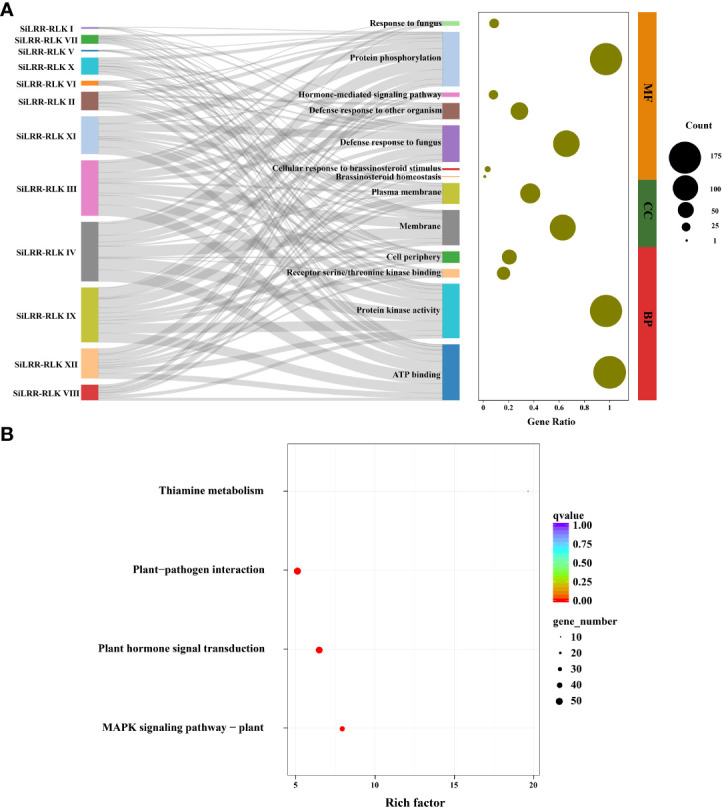
GO annotation and KEGG enrichment of SiLRR-RLK proteins. **(A)** GO annotation of 12 SiLRR-RLK protein subfamilies. MF, Molecular Function; CC, Cellular Component; BP, Biological Process. **(B)** KEGG enrichment of SiLRR-RLK proteins.

To further explore the potential functions of SiLRR-RLKs, GO (Gene Ontology) annotation was carried out. The results showed that SiLRR-RLKs were mainly involved in phosphorylation and defense response to fungus in terms of biological processes and functioned in kinase activity and ATP binding in molecular function (**Figure 3A**). All SiLRR-RLKs were predicted to localize to the membrane or plasma membrane. Consistently, all SiLRR-RLKs showed the characteristics of a high aliphatic index and low hydrophilicity (**Supplementary Table S2**), which further supported the idea that they were located on the plasma membrane. KEGG (Kyoto Encyclopedia of Genes and Genomes) enrichment of SiLRR-RLKs illustrated that SiLRR-RLKs were mainly enriched in thiamine metabolism, MAPK signaling pathway, plant hormone signal transduction and plant−pathogen interaction (**Figure 3B**). All these results suggest that SiLRR-RLK proteins are essential for signaling recognition and transduction in the stress resistance, growth and development of sesame.

### 3.3 *Cis*-element analysis of SiLRR-RLK genes

**Figure 4:**
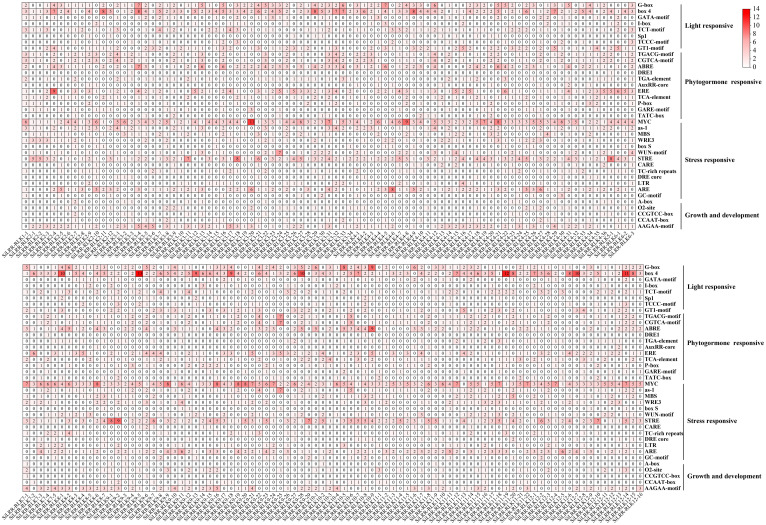
The distribution of *cis*-elements in *SiLRR-RLK* promoters. The gradient colors represent the number of cis-elements in *SiLRR-RLK* promoters.

Gene promoters in plants can regulate the expression of genes to respond to different biotic or abiotic stresses and different growing environments, hence, assessment of cis-elements and transcription factor binding sites in promoters is crucial for understanding transcriptional regulation and gene function (Biłas et al., 2016). The upstream sequences (~2000 bp) of the promoter were obtained to confirm the expression features of *SiLRR-RLKs.* The cis-elements of the *SiLRR-RLK* promoters were explored using the PlantCARE database (Lescot et al., 2002). The detailed effects of these motifs (cis-elements) are presented in **Supplementary Table S3**. *SiLRR-RLK* promoters contained many cis-elements in response to stresses, illustrating their potential roles in plant responses to adverse environments. *Cis*-elements of *SiLRR-RLK* promoters include light-responsive elements, phytohormone-responsive elements, stress-responsive elements and growth and development elements (**Figure 4**). The most abundant element in *SiLRR-RLK* promoters was the Box4 (Light) element, followed by the MYC (Drought) and STRE (Stress) elements (**Figure 4**; **Supplementary Table S3**), illustrating that *SiLRR-RLKs* not only had an important role in the light response but were also crucial in the response to both biotic and abiotic stresses. In addition, *SiLRR-RLKs* possessed the most AAGAA-motif (Seed specific expression) and ERE (Ethylene) elements in terms of growth development and hormone response.

Transcription factors (TFs) play key roles in many biological processes by regulating the expression of target genes. To investigate the possible regulatory relationship between TFs and *SiLRR-RLK* genes, the TF binding site prediction on PlantTFDB (Jin et al., 2017) was used. The results showed that the *SiLRR-RLK* genes could be regulated by 37 TF families (**Supplementary Figure S1**, **Supplementary Table S4**). C2H2, MIKC_MADS, MYB, AP2 and Dof were the TFs that can regulate most *SiLRR-RLK* genes. These TF families are involved in almost every aspect of plant development, hormone signaling, plant defense and stress response, suggesting that *SiLRR-RLKs* are extensively involved in the growth and stress defense of sesame.

### 3.4 Duplication and syntenic analysis of SiLRR-RLK genes

**Figure 5:**
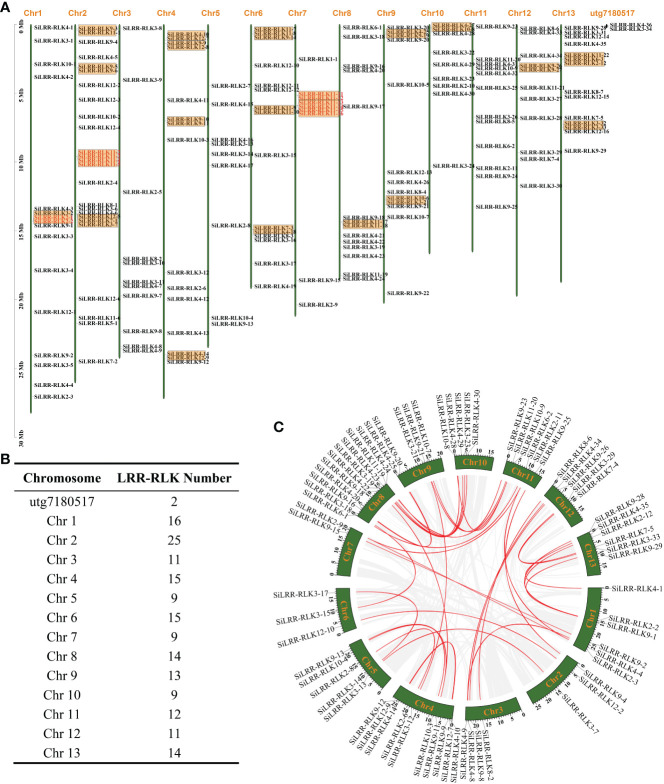
Chromosomal arrangement and gene duplication of *SiLRR-RLK* genes. **(A)** 173 *SiLRR-RLK* genes were mapped to 13 sesame chromosomes while 2 *SiLRR-RLK* genes mapped to unanchored scaffolds. The orange box indicates a gene cluster and the red names represent tandem duplication genes. **(B)** Number of *SiLRR-RLK* genes on each sesame chromosome. **(C)** The segmental duplication gene pairs of *SiLRR-RLK* genes. The gray lines indicate all the segmental duplicated gene pairs while red lines highlight the *SiLRR-RLK* pairs within the sesame genome.

We found that 173 *SiLRR-RLKs* were unevenly distributed on 13 sesame chromosomes (Chr) while *SiLRR-RLK3-34* and *SiLRR-RLK4-36* were distributed on an unanchored scaffold (**Figure 5A**). Twenty-five *SiLRR-RLK* genes were mapped to Chr 2, followed by 15 *SiLRR-RLK* genes on Chr 4 and Chr 6. In contrast, minimum *SiLRR-RLK* genes (9) was found on Chr 5, Chr 7 and Chr 10 (**Figure 5B**). A total of 48 *SiLRR-RLK* genes formed 21 gene clusters. Chr 2 and Chr 4 both had maximum gene clusters with four. There are 2-4 *SiLRR-RLK* members within the gene clusters, most of them contain 2 *SiLRR-RLK* genes. The tandem duplication *SiLRR-RLK* genes were identified in each cluster with a threshold of 70% sequence similarity between two aa sequences of *SiLRR-RLKs*. Of the 21 gene clusters, 12 *SiLRR-RLK* genes from 7 clusters were considered to be tandem duplicated gene pairs (**Figure 5A**). In addition, segmental duplication of *SiLRR-RLK* genes was further analyzed within the sesame genome. 38 segmentally duplicated *SiLRR-RLK* pairs made by 70 *SiLRR-RLK* genes were predicted within the sesame genome (**Figure 5C**). A total of 46.29% of *LRR-RLK* genes underwent tandem or segmental duplication events, implying that gene duplication events were momentous in the expansion of the *SiLRR-RLK* gene family.

### 3.5 Evolution analysis of SiLRR-RLK genes in several plants

**Figure 6:**
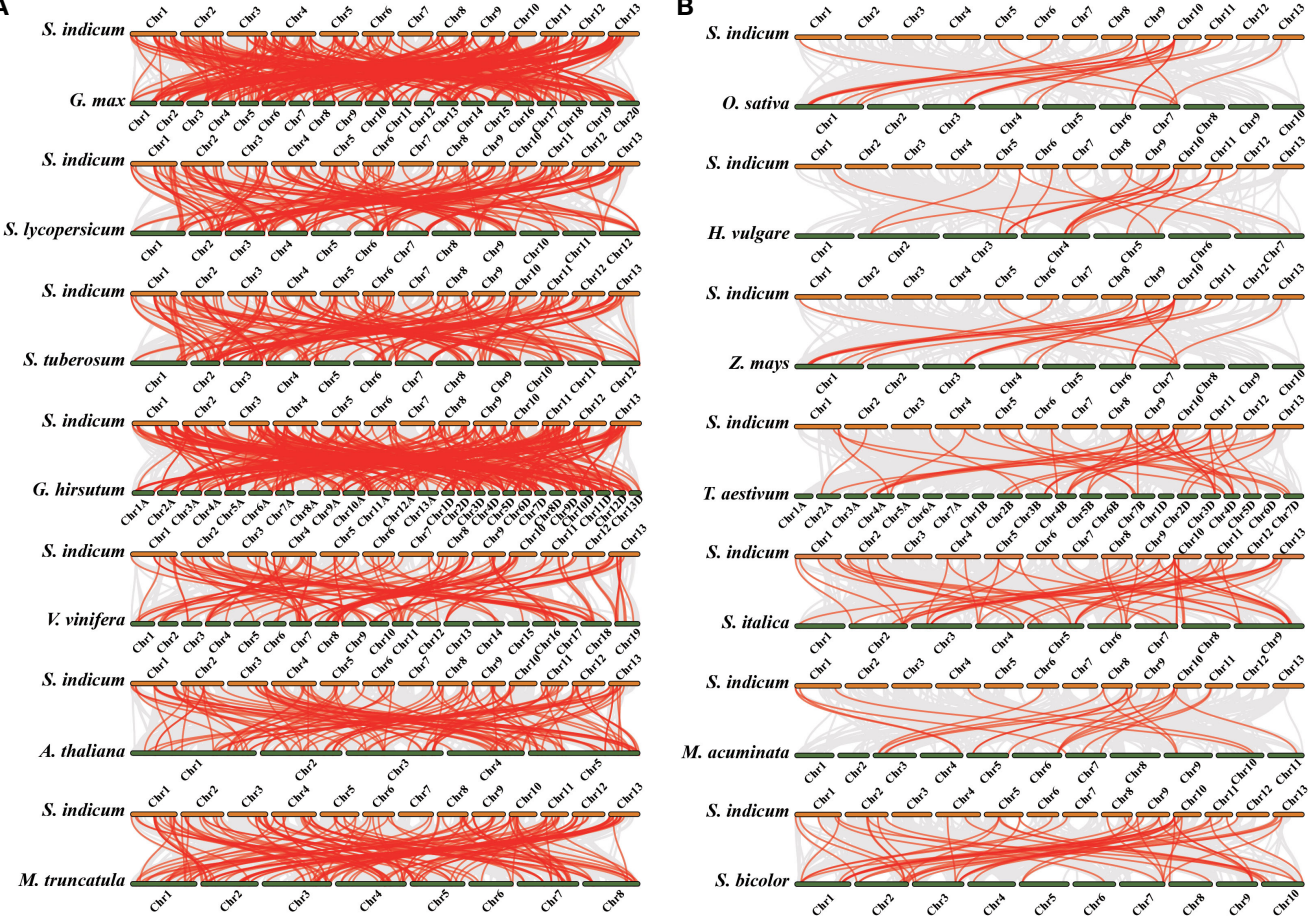
Synteny analysis of *LRR-RLK* genes between *S. indicum* and other plant species. The gray lines indicate all the syntenic gene pairs while red lines highlight the *SiLRR-RLK* pairs between *S. indicum* and other plant species. **(A)** Synteny analysis of *LRR-RLK* genes between *S. indicum* and dicotyledonous plants. **(B)** Synteny analysis of *LRR-RLK* genes between *S. indicum* and monocotyledonous plants.

To infer the syntenic relationship of *LRR-RLK* genes in several plants, seven dicotyledons (*G. max*, *S. lycopersicum*, *S. tuberosum*, *G. hirsutum*, *V. vinifera, A. thaliana* and *M. truncatula*) (**Figure 6A**) and seven monocotyledons (*O. sativa*, *H. vulgare*, *Z. mays*, *T. aestivum*, *S. italica, M. acuminata* and *S. bicolor*) (**Figure 6B**) were used for evolution analysis with *S. indicum*. The *LRR-RLK* genes are homologous to genes in the dicotyledonous reference plants, and the number of homologous *LRR-RLK* genes is 120 (*G. max*), 132 (*S. lycopersicum*), 131 (*S. tuberosum*), 114 (*G. hirsutum*), 113 (*V. vinifera*), 98 (*A. thaliana*) and 114 (*M. truncatula*) (**Supplementary Table S5**). Nonetheless, only 36 (*O. sativa*), 16 (*H. vulgare*), 14 (*Z. mays*), 20 (*T. aestivum*), 38 (*S. italica*), 16 (*M. acuminata*) and 41 (*S. bicolor*) homologous *LRR-RLK* genes existed in monocotyledons (**Supplementary Table S5**). More homologous *LRR-RLK* genes were found in dicotyledons than in monocotyledons. In addition, *SiLRR-RLK4-27* and *SiLRR-RLK10-9* were homologous with all 14 species, suggesting that they are crucial in the evolution of the *LRR-RLK* gene family. Notably, *SiLRR-RLK10-9* also underwent segmental replication events in sesame (**Figure 5C**).

To further investigate the evolutionary relationship of LRR-RLK in dicotyledons, phylogenetic analysis of LRR-RLK proteins in dicotyledons was performed (**Supplementary Figure S2**). The results showed that SiLRR-RLK tended to gather with the LRR-RLKs of *A. thaliana* and *S. tuberosum*. We also used the MEME website to search for 10 conserved motifs of all LRR-RLK proteins. We found that the LRR-RLKs in *S. indicum* shared the most similar motif compositions with *A. thaliana* and *S. tuberosum* in the same branch, suggesting that SiLRR-RLKs were more closely related to those of *A. thaliana* and *S. tuberosum*.

### 3.6 *In silico* expression profiles of SiLRR-RLK genes in diverse tissues

**Figure 7:**
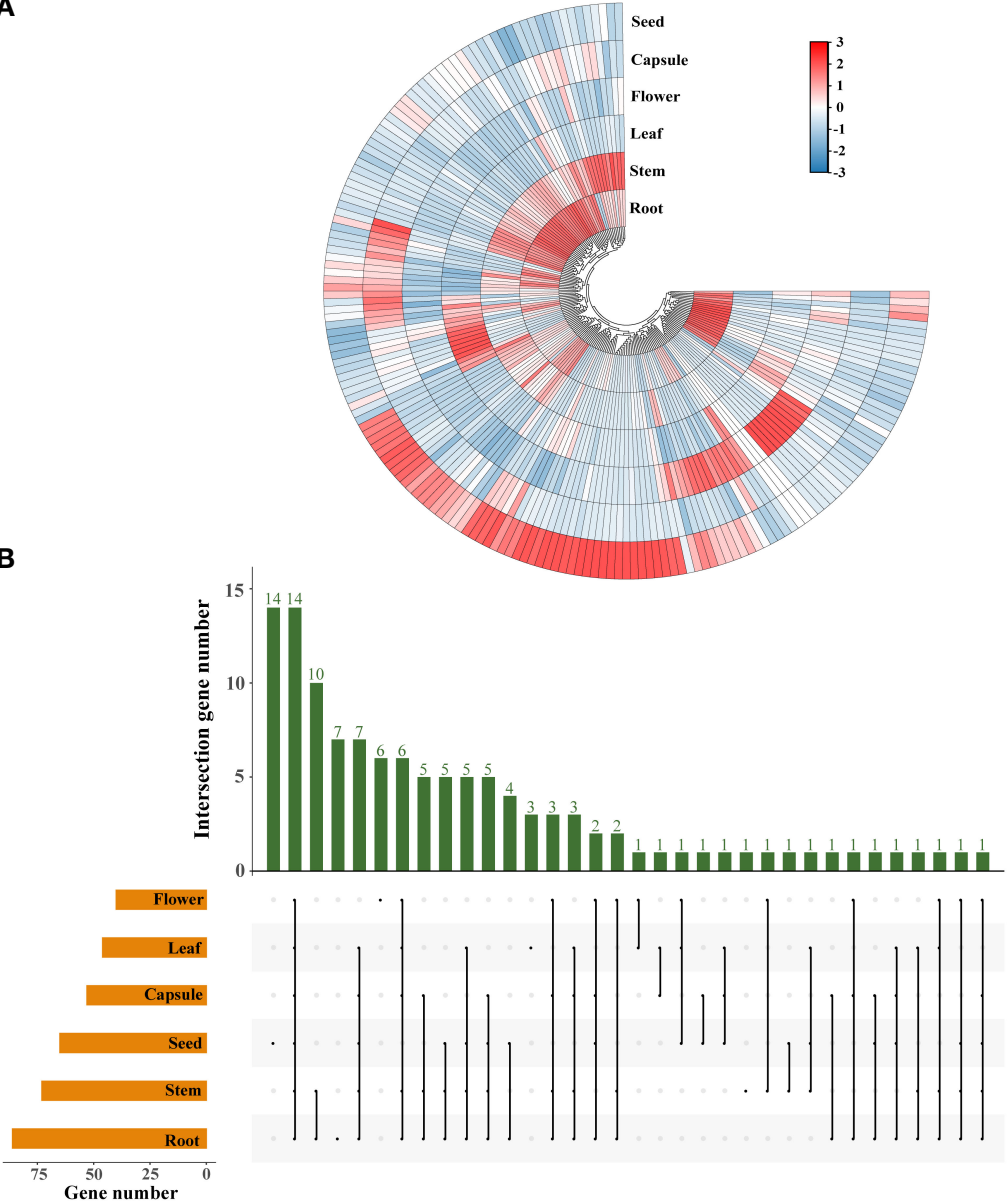
Expression profiles of *SiLRR-RLK* genes in different tissues. **(A)** Tissue-specific expression of *SiLRR-RLK* genes. The color scale shows the range of normalized FPKM values. **(B)** Number of *SiLRR-RLK* genes highly expressed in each tissue. Orange bars show the *SiLRR-RLK* genes highly expressed in each tissue while green bars indicate *SiLRR-RLK* genes highly expressed across diverse tissues.

To gain a broader understanding of the functions of *SiLRR-RLKs*, we analyzed the divergence in spatial expression among *SiLRR-RLK* genes. Most *SiLRR-RLKs* exhibited different expression patterns in different tissues (**Figure 7A**). Some *SiLRR-RLK* genes were expressed tissue-characteristically. For instance, *SiLRR-RLK8-5* and *SiLRR-RLK12-9* were found to be expressed only in seeds, *SiLRR-RLK3-13* and *SiLRR-RLK4-6* were found to be expressed only in flowers, *SiLRR-RLK11-5* and *SiLRR-RLK11-18* were found to be expressed only in roots (*SiLRR-RLK* genes with FPKM values less than 0.1 were not considered expressed) (**Supplementary Table S6**). The gene expression patterns provided a preliminary clue to its function. 86, 73, 46, 40, 53 and 65 *LRR-RLK* genes were highly expressed (*SiLRR-RLK* genes with FPKM value more than 10 were considered as expressed highly) in roots, stems, leaves, flowers, capsules and seeds, respectively (**Figure 7B**). Of note, 14 *SiLRR-RLKs* (*SiLRR-RLK3-30*, *SiLRR-RLK3-16*, *SiLRR-RLK3-10*, *SiLRR-RLK3-5*, *SiLRR-RLK10-1*, *SiLRR-RLK3-22*, *SiLRR-RLK8-6*, *SiLRR-RLK6-3*, *SiLRR-RLK8-2*, *SiLRR-RLK3-26*, *SiLRR-RLK3-7*, *SiLRR-RLK9-8*, *SiLRR-RLK9-14* and *SiLRR-RLK3-1*) exhibited constitutively high expression across different tissues, indicating their important roles in the growth and development of sesame (**Figure 7B**). For example, AtTMK1 (Transmembrane kinase 1), a homolog of SiLRR-RLK3-16, can active GTPase in auxin sensing (Cao et al., 2019). TMK1-mediated auxin signaling regulates membrane-associated clathrin in *Arabidopsis* roots (Wang et al., 2022), suggesting the importance of SiLRR-RLK3-16 in sensing auxin.

The AAGAA motif (seed-specific expression) was found to be the most abundant cis-element in the promoter of *SiLRR-RLK* in terms of growth and development. Therefore, the expression pattern of *SiLRR-RLK* in seed development was determined based on PRJNA739094 (Zhang et al., 2021) (**Supplementary Figure S3**, **Supplementary Table S7**). The results showed that most *SiLRR-RLK* genes had a higher expression level in the early stage (S1 and S2) of seed development and then decreased in the later stage (S3 and S4), suggesting potential effects of *SiLRR-RLK* genes in early seed development. Notably, *SiLRR-RLK3-4*, *SiLRR-RLK3-6*, *SiLRR-RLK3-17* and *SiLRR-RLK9-16* possessed the most AAGAA motifs with five (**Figure 4**). Among these, *SiLRR-RLK3-6* expressed at a low level in all stages. *SiLRR-RLK3-4* were induced at early stages S1, S2 and S3. Likewise, *SiLRR-RLK3-17* and *SiLRR-RLK9-16* were highly expressed during early seed development S1 and S2, implying that *SiLRR-RLK3-4*, *SiLRR-RLK3-17* and *SiLRR-RLK9-16* might contribute to sesame seed development. Furthermore, we found several *SiLRR-RLKs* (*SiLRR-RLK3-1*, *SiLRR-RLK3-7*, *SiLRR-RLK6-3* and *SiLRR-RLK10-4*) that were highly expressed during whole seed development (FPKM>10), which may also function in sesame seed development.

### 3.7 *In vitro* expression pattern of SiLRR-RLK genes in response to phytohormone treatment

**Figure 8:**
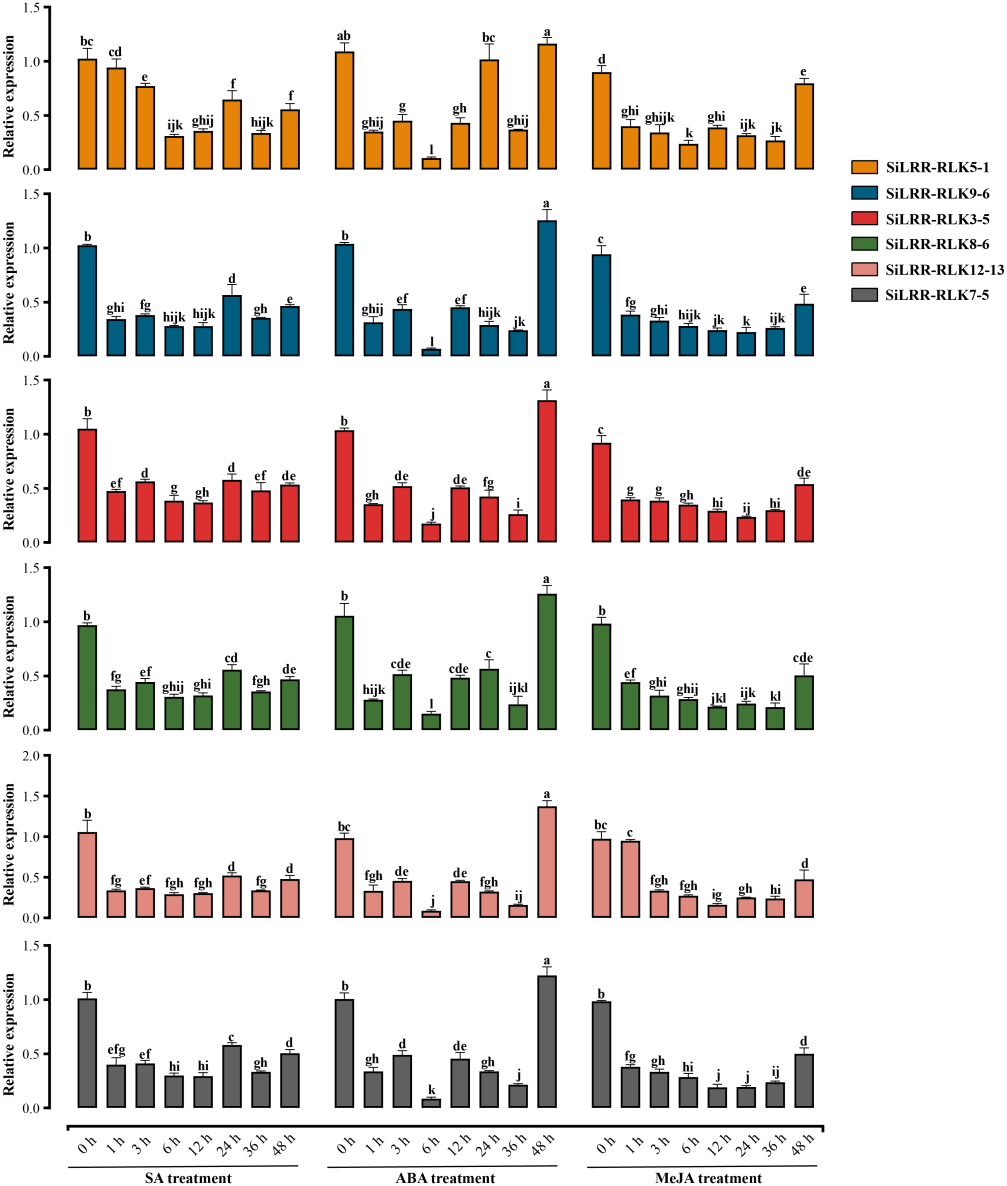
Relative expression level of *SiLRR-RLK5-1* (Homolog of SOBIR1), *SiLRR-RLK9-6* (Homolog of RLK7), *SiLRR-RLK3-5* (Homolog of BAK1), *SiLRR-RLK8-6* (Homolog of BRI1), *SiLRR-RLK12-13* (Homolog of PEPR1) and *SiLRR-RLK7-5* (Homolog of PSKR1) genes at 0 h, 1 h, 3 h, 6 h, 12 h, 24 h, 36 h and 48 h post treated by SA, ABA and MeJA.

*LRR-RLK* genes were functioned in plant hormone signaling pathways. Therefore, we selected *SiLRR-RLK5-1* (Homolog of SOBIR1), *SiLRR-RLK9-6* (Homolog of RLK7), *SiLRR-RLK3-5* (Homolog of BAK1), *SiLRR-RLK8-6* (Homolog of BRI1), *SiLRR-RLK12-13* (Homolog of PEPR1) and *SiLRR-RLK7-5* (Homolog of PSKR1) genes to investigate their expression patterns under SA, ABA and MeJA treatments (**Figure 8**).

The *SiLRR-RLK9-6*, *SiLRR-RLK3-5*, *SiLRR-RLK8-6*, *SiLRR-RLK12-13* and *SiLRR-RLK7-5* genes were down-regulated significantly at 1 h post SA treatment and remained suppressing within 48 h post SA treatment. However, *SiLRR-RLK5-1* was significantly down-regulated expression at 3 h post SA treatment. It is suggested that these genes play a negative role in the early stage (48 h) in SA signaling pathway. Under ABA treatment, *SiLRR-RLK5-1, SiLRR-RLK9-6, SiLRR-RLK3-5, SiLRR-RLK8-6, SiLRR-RLK12-13* and *SiLRR-RLK7-5* genes were significantly down-regulated at 1h and decreased to the lowest level at 6 h, followed by a significant up-regulation of expression at 48 h (*SiLRR-RLK3-5* restored its expression level at 48 h). Under MeJA treatment, *SiLRR-RLK5-1, SiLRR-RLK9-6, SiLRR-RLK3-5, SiLRR-RLK8-6, SiLRR-RLK12-13* and *SiLRR-RLK7-5* genes were significantly down-regulated, followed by partial restoration of their expression levels at 48 h. Notably, the expression trends of these six genes were similar under phytohormone treatment, suggesting that they may form dimers or polymers in phytohormone signaling pathway and synergistically regulate the downstream pathways.

### 3.8 *In silico* integrative expression analysis of SiLRR-RLKs during *M. phaseolina* stress

**Figure 9:**
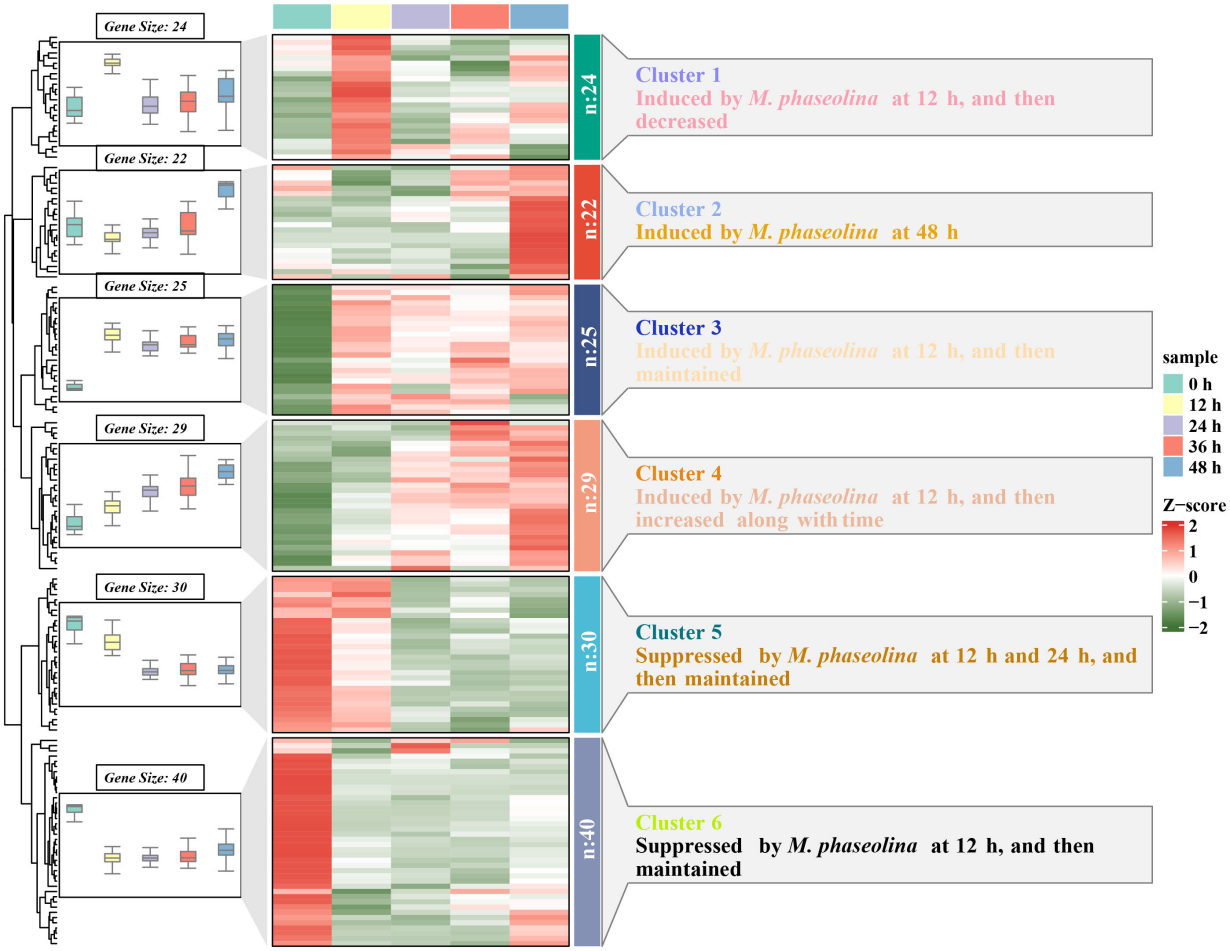
Expression patterns of *SiLRR-RLK* genes under *M. phaseolina* stress (0 - 48 h). The box plot on the left represents the expression trend of *SiLRR-RLK* genes in the same cluster, the heatmap in the middle indicates the expression patterns of *SiLRR-RLK* genes, and the annotation on the right explains the expression trend of *SiLRR-RLK* genes in each cluster.

LRR-RLKs act as cell surface receptors and play a crucial role in signal sensing and transduction. To unravel the function of SiLRR-RLKs in response to pathogen *M. phaseolina*, the expression patterns of *SiLRR-RLK* genes under stress were investigated (**Figure 9**; **Supplementary Table S8**). Considering that there are many *LRR-RLK* genes in sesame, we divided them into six clusters based on their expression patterns (FPKM>0) (**Supplementary Figure S4**). The results showed that the *SiLRR-RLK* genes in Cluster 5 and Cluster 6 were decreased after inoculation with *M. phaseolina*, they may mediate the susceptibility in plant immunity (**Figure 9**; **Supplementary Figures S4E, S4F**). However, most *SiLRR-RLKs* were induced at different times in Cluster 1, Cluster 2, Cluster 3 and Cluster 4 under *M. phaseolina* stress, which further confirmed the widely known disease resistance of *LRR-RLKs* (**Figure 9**; **Supplementary Figures S4A-D**). For instance, *SiLRR-RLK11-10* in Cluster 1 were upregulated 4.68-fold at 12 hours after infection while *SiLRR-RLK12-6* and *SiLRR-RLK9-22* in Cluster 2 were upregulated 4.89- and 10.48-fold at 48 hours after infection, respectively. *SiLRR-RLK* genes in Cluster 3 were upregulated 2-3 times overall during *M. phaseolina* treatment. Moreover, the expression of *SiLRR-RLK* genes in Cluster 4 were continuously induced by *M. phaseolina* (**Supplementary Figures S4A-D**, **Supplementary Table S8**).

**Figure 10:**
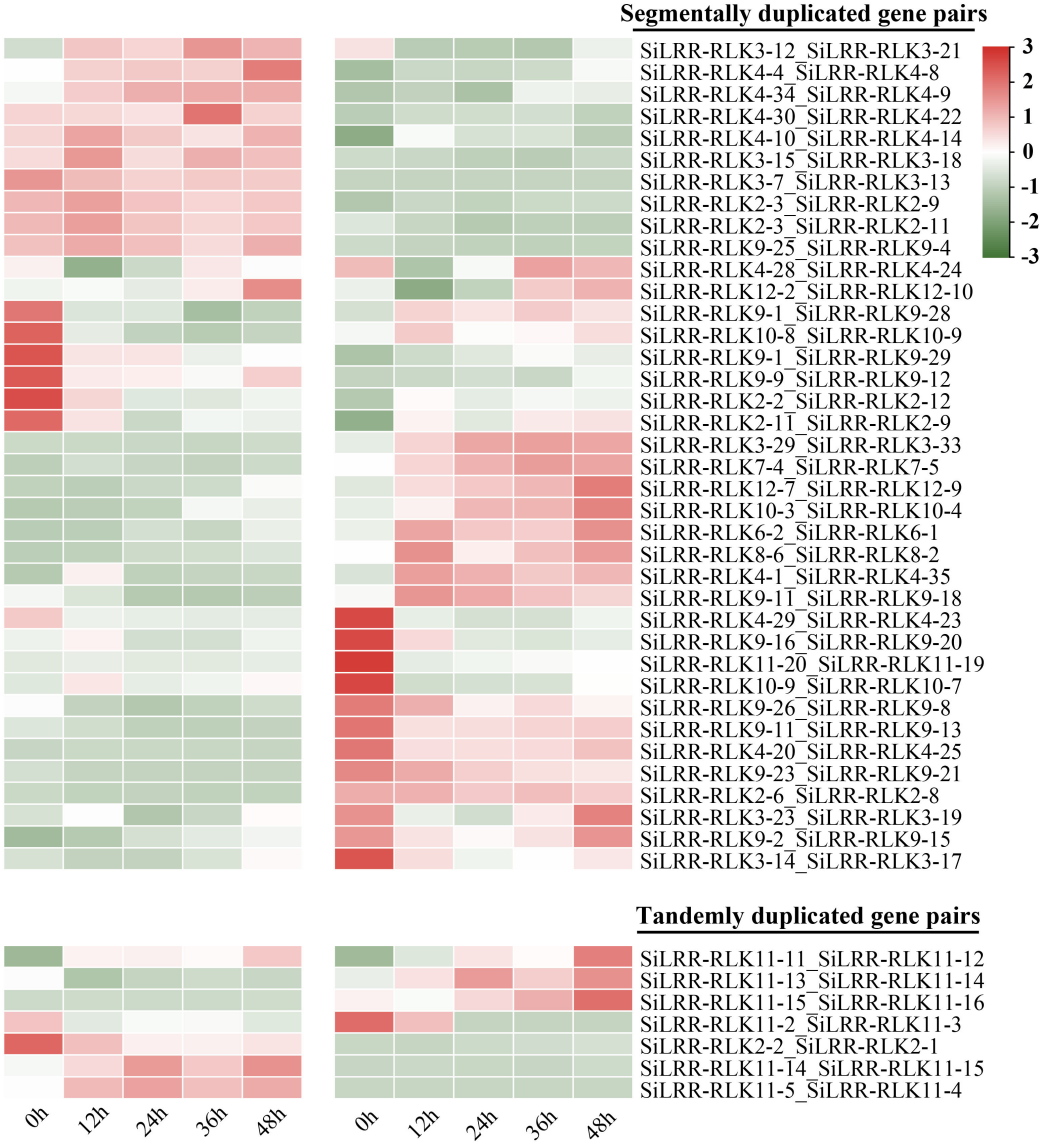
Expression patterns of tandemly and segmentally duplicated *SiLRR-RLK* gene pairs during *M. phaseolina* infection (0 - 48 h). “_” represents two *SiLRR-RLKs* are duplicated gene pairs.

The duplicated *SiLRR-RLK* genes identified were analyzed synchronously with their expression patterns to identify the genes designated for novel functions. The expression patterns of 7 pairs of tandemly duplicated *SiLRR-RLK* genes and 38 pairs of segmentally duplicated *SiLRR-RLK* genes during *M. phaseolina* stress were analyzed in a heatmap (**Figure 10**). We found that most tandemly and segmentally duplicated *SiLRR-RLK* genes exhibited antagonistic expression profiles under *M. phaseolina* stress, suggesting a function of redundancy between *SiLRR-RLK* genes during sesame disease resistance (**Figure 10**). There were only a few exceptions, the *LRR-RLK11-11*:*LRR-RLK11-12* gene pair showed a similar expression profile under *M. phaseolina* stress, that is, they were both induced at post infection.

### 3.9 Molecular protein−protein interaction network of SiLRR-RLKs

**Figure 11:**
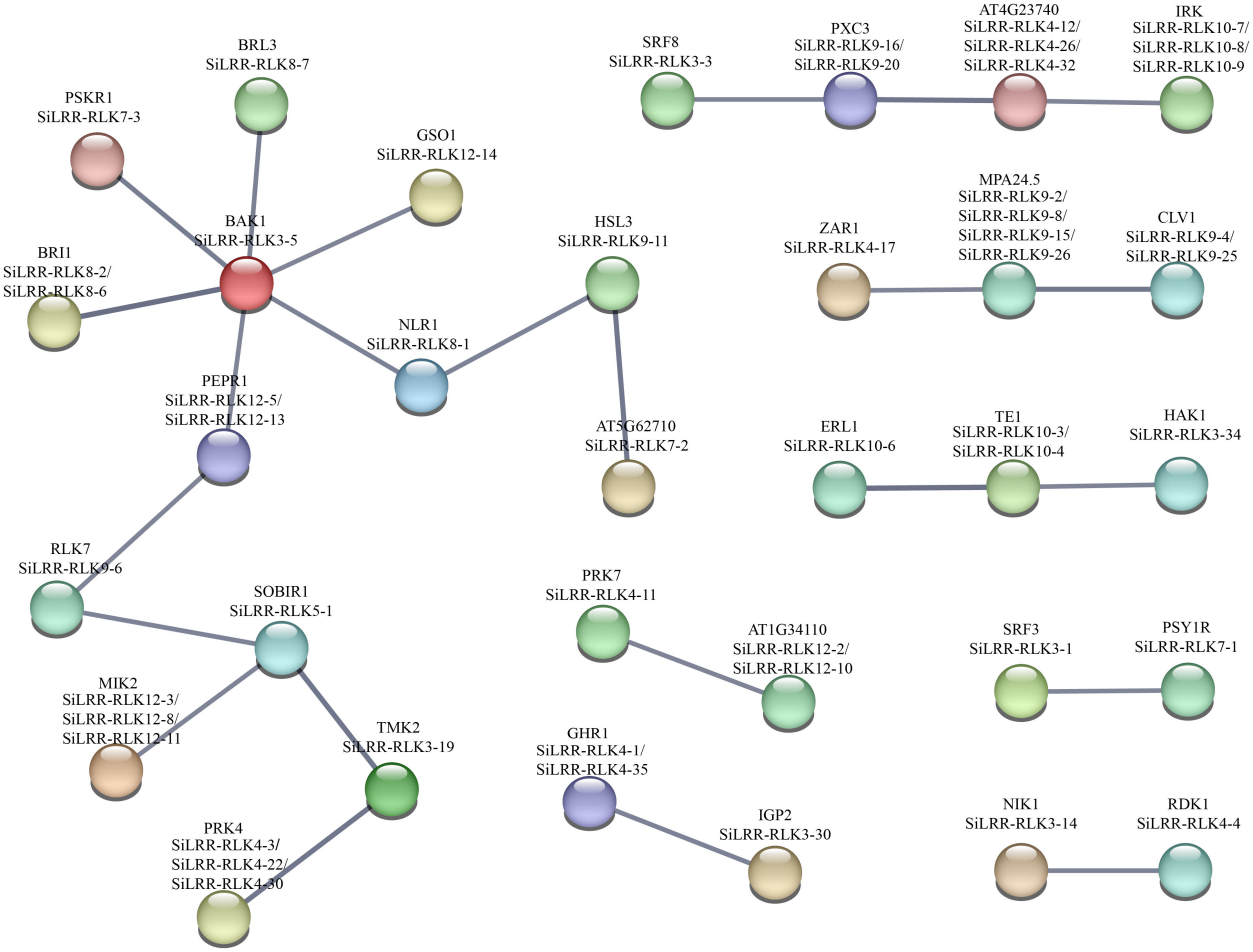
A protein−protein interaction network for SiLRR-RLKs based on their orthologs in *Arabidopsis*. SiLRR-RLK proteins are shown in brackets with *Arabidopsis* orthologs.

According to well-studied investigations, there are many complex interactions within the *LRR-RLK* gene family. To further gain insight into the functions of the SiLRR-RLK proteins, we constructed a PPI network by STRING database (https://STRING-db.org/) based on the well-studied LRR-RLKs in *Arabidopsis*. As shown in **Figure 11**, the SiLRR-RLK members showed interactions with some other members.

Obviously, SiLRR-RLK3-5, the homolog of AtBAK1, is the hub functional gene in the PPI network. Studies have showed that AtBAK1, acting as a coreceptor with other proteins, can form complexes (Dimer, trimer or tetramer) such as SOBIR1/BAK1, BAK1/BIR1, ER/BAK1/TMM, BIK1/BAK1/ERL1/ERL2, FLS2/BAK/BIK1 and FLS2/BIK1/RBOHD, and these complexes are all important in relaying signals to downstream components in plant immunity system (Gao et al., 2009; Lu et al., 2010; Wang et al., 2011; Li et al., 2014; Lin et al., 2014; Jordá et al., 2016). In addition, other AtLRR-RLK genes in the network have also been proven to be involved in plant biotic stress, such as SOBIR1, a homolog of SiLRR-RLK5-1, which was reported to form a complex with BAK1 for immunity against the fungi *Phytophthora infestans* and *Sclerotinia sclerotiorum* (Gao et al., 2009; Liu et al., 2016). SiLRR-RLK4-17 is homologous to ZAR1, which is a calcium-permeable channel triggering plant immune signaling (Bi et al., 2021). Notably, SiLRR-RLK8-6, a homolog of AtBRI1, was highly expressed in all tissues. BRI1 acts as a BR receptor and is extensively involved in plant growth, development and stresses (Wang et al., 2001). Recent studies have shown that BRI1 can form a heterodimer with SAUR15, which activates the plasma membrane H^+^-ATPase to promote *Arabidopsis* organogenesis (Li et al., 2022). In addition, BRI1 is another LRR-RLK that can bind to BAK1, and the BRI1/BAK1 complex regulates stem elongation, vascular differentiation, seed size, fertility, flowering time and senescence by BR signaling in *A. thaliana* (Li et al., 2002; Nam and Li, 2002; Wang et al., 2005). Notably, BRI1 in cereals has been shown to contribute to disease resistance (Goddard et al., 2014) and drought tolerance (Feng et al., 2015) in plants, indicating that SiLRR-RLKs have complex biological functions by participating in the crosstalk between plant growth and development and stress.

### 3.10 Coexpression analysis of LRR-RLK genes in response to *M. phaseolina*

**Figure 12:**
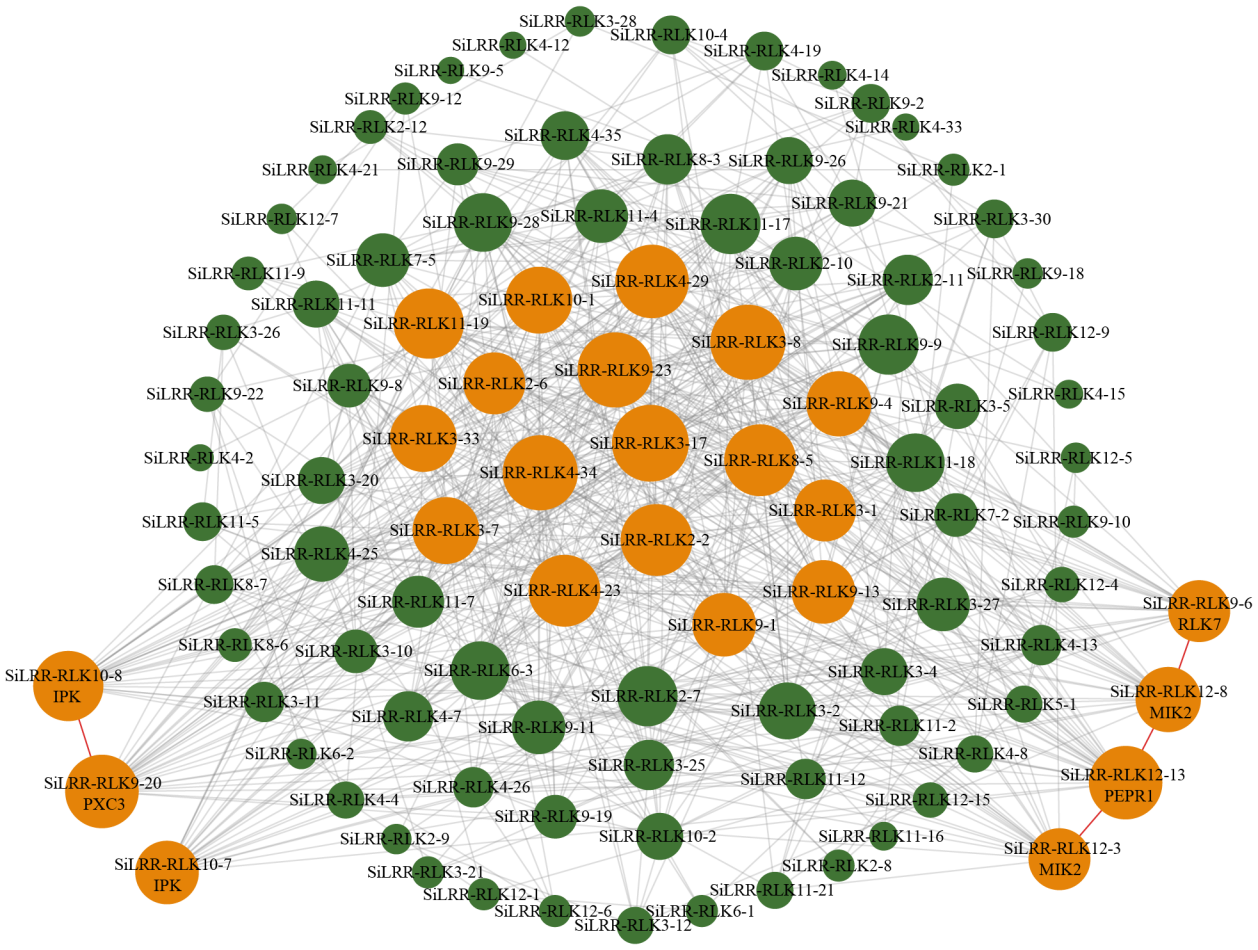
Coexpression network of *SiLRR-RLK* genes in response to *M. phaseolina*. The larger nodes represent core *SiLRR-RLKs* in the network, while the smaller nodes represent noncore *SiLRR-RLKs*. The size of the node circle is positively correlated with the number of *SiLRR-RLKs* it interacts. The orange nodes indicate core *SiLRR-RLKs* that may form multimers to defend against *M. phaseolina*.

Based on the sequence structure, functional annotation, expression patterns and PPI prediction of SiLRR-RLKs, we can conclude that SiLRR-RLKs are crucial for plant immunity. In addition, previous studies have shown that SiLRR-RLKs are vital components in response to *M. phaseolina* stress (Yan et al., 2021). To further understand the relationship between *SiLRR-RLK* genes and sesame disease resistance, the expression patterns of *SiLRR-RLK* genes under *M. phaseolina* stress were used for correlation analysis. *SiLRR-RLK* gene pairs with a Pearson correlation coefficient greater than 0.95 or less than -0.95 suggested a correlation between the two *SiLRR-RLK* genes. The coexpression network of *SiLRR-RLK* genes was constructed according to the relationship between *SiLRR-RLK* genes (**Figure 12**). In a coexpression network, most genes interact with only a few other genes, while a few interact with a large number of other genes, which are the core genes in this gene network. Core *SiLRR-RLK* genes in the coexpression network might be vital in sesame resistance to *M. phaseolina*. Additionally, two core gene sets in the network attracted our attention (**Figures 11**, **12**). One is the BAK1/PEPR1/RLK7/SOBIR1/MIK2 signaling pathway, whose function in plant immunity has been well elucidated in other plants. The other is the SRF8/PXC3/IRK signaling pathway. Notably, there exist a correlation between SiLRR-RLK10-8 and SiLRR-RLK9-20, as well as SiLRR-RLK12-3, SiLRR-RLK12-13, SiLRR-RLK12-8 and SiLRR-RLK9-6 (**Figures 11**, **12**). Combined with coexpression analysis and the PPI network, the core genes, *SiLRR-RLK12-3/SiLRR-RLK12-13/SiLRR-RLK12-8/SiLRR-RLK9-6* and *SiLRR-RLK10-8/SiLRR-RLK9-20*, may be the core components of disease resistance to *M. phaseolina* in sesame.

## 4 Discussion

Recently, the *LRR-RLK* gene family has been identified in many plant species, and the number of *LRR-RLK* family members varies greatly. The proportion of *SiLRR-RLK* genes was consistent with Liu et al. (Liu et al., 2017), which showed a 0.67-1.39% proportion in angiosperm species. In higher plants, the number of identified *LRR-RLK* genes ranged from 180 (*C. sativus*) to 589 (*T. elongatum*) (Soltabayeva et al., 2022). A recent study has identified 14 classes resistance (R) genes, including *LRR-RLK* subclass, in the sesame genome (Miao et al., 2023). In this study, 175 *SiLRR-RLK* genes were identified from the sesame genome, accounting for 0.73% of the sesame genome and 14.61% of sesame R genes. Although there is a lack of information on the function of *LRR-RLK* genes in sesame, the evolutionary diversity and function of *SiLRR-RLK* genes can be inferred from phylogenetic analysis, protein structure, gene structure and expression profiles. The phylogenetic tree revealed that SiLRR-RLKs can be divided into 12 subfamilies. SiLRR-RLKs occurred in almost every major branch together with the *Arabidopsis* LRR-RLK subfamily (**Figure 1**), indicating that all *Arabidopsis* LRR-RLK subfamilies share a common ancestor with sesame. Additionally, the collinearity analysis showed that the homologous *LRR-RLK* genes existed much more in dicotyledons than monocotyledons (**Figure 6**). It is implied that the duplication of the *LRR-RLK* gene probably occurred after the differentiation of dicotyledons and monocotyledons, which has been consistent with a previous investigation(Miao et al., 2023).

Segmental and tandem replication are important drivers of the expansion of gene families, especially in the evolution of plant *LRR-RLK* gene families (Lehti-Shiu et al., 2009; Lehti-Shiu and Shiu, 2012). A total of 420 (71.31%) *LRR-RLK* genes with replication events were detected in *T. ponticum*, involving 191 segmentally duplicated *SiLRR-RLK* pairs and 145 tandemly duplicated *SiLRR-RLK* pairs (Mishra et al., 2021). It has been found that 73.3% and 20.3% of *LRR-RLK* genes in soybean were involve in segmental duplication and tandem duplication (Zhou et al., 2016). Similarly, in the present study, 81 (46.29%) *SiLRR-RLK* genes involved in 38 segmental pairs and 7 tandem pairs were perceived (**Figure 5**). Therefore, it is inferred that the expansion of the *LRR-RLK* gene family is mainly caused by gene segmental duplication. Interestingly, *SiLRR-RLK10-9* was found to have collinearity with other 14 species (**Figure 6**), and *SiLRR-RLK10-9* also underwent segmental replication events in sesame, suggesting its key contributions to the expansion of the *SiLRR-RLK* gene family.

The *SiLRR-RLK* IV and III subfamilies, representing the two largest subfamilies, exhibited duplication events. *SiLRR-RLKs* in the IV and III subfamilies were also syntenic with the *LRR-RLKs* in the other 14 species (**Supplementary Table S5**). 36 and 34 *LRR-RLK* members from sesame were found to form the *SiLRR-RLK* IV and III subfamilies, respectively, based on the phylogenetic tree (**Figure 1**). Furthermore, there were 16 genes and 12 genes underwent segmental duplication in IV and III subfamilies, respectively (**Figures 1**, **5C**). Under *M. phaseolina* stress, 13 and 16 *SiLRR-RLK* genes in IV and III subfamilies were induced significantly (**Figure 10**, **Supplementary Table S8**). Notably, proteins from subfamilies IV and III were both assigned to the GO terms phosphorylation and kinase activity (**Figure 3A**). These results point to the idea that the duplicated events within the *SiLRR-RLK* IV and III subfamilies during evolution may contribute to the perception of *M. phaseolina* stress signals in sesame.

The *LRR-RLK* genes are crucial in recognition and signal transduction in biotic and abiotic stresses, as shown by the fact that their promoters possess many phytohormone and stress responsive cis-elements (**Figure 4**), hence, the ever-changing environment may also lead to the replication and expansion of the *SiLRR-RLK* gene family. On the other hand, evidence that there are the ubiquitous redundant functions of *SiLRR-RLK* genes has indicated that the diversity of *SiLRR-RLK* genes may also be the result of random genomic drift (Eyüboglu et al., 2007; Albrecht et al., 2008). After duplication, duplicated genes usually accumulate mutations and lead to a functional diversification of LRR-RLK proteins. *Arabidopsis* LRR-RLKs are primarily involved in regulating plant growth and development and stress responses (Biotic and abiotic stresses) or both (Li and Tax, 2013). In this investigation, KEGG enrichment of SiLRR-RLKs revealed the pathways involved in resistance to *M. phaseolina*, such as MAPK signaling pathway, plant hormone signal transduction and plant−pathogen interaction pathway (**Figure 3B**). SiLRR-RLKs may sense extracellular signals, act as early warning genes, and then regulate the early stress response in plants (Lin et al., 2017). In this study, SiLRR-RLKs were shown to play a role in plant−pathogen interactions by transmitting and amplifying signals downstream through protein phosphorylation and kinase activity (**Figure 3**).

In general, the expression patterns of genes represent their potential functions. Therefore, the expression profiles of *SiLRR-RLKs* under *M. phaseolina* stress may help us to gain insight on the function of *SiLRR-RLKs*. In our research, several genes in response to fungal infection were identified. Many *SiLRR-RLKs* act as pattern recognition receptors to initiate the PTI (Pathogen-associated molecular pattern-triggered immunity). SiLRR-RLK3-5, homologous to AtBAK1, can be continuously induced during *M. phaseolina* infection (**Supplementary Table S8**), implicating its positive function during plant immunity, which is supported by the central position of SiLRR-RLK3-5 in the PPI network (**Figure 11**). In *Arabidopsis*, the BAK1/BRI1 complex regulates the cell death process to participate in the immune response by the BR signaling pathway (Li et al., 2002). Likewise, in tobacco, NbBRI1 participates in brassinosteroid-regulated immune responses by regulating the concentrations of H_2_O_2_ and NO (Deng et al., 2016). In sesame, SiLRR-RLK8-6, which is homologous to BRI1, were induced during *M. phaseolina* stress (**Supplementary Table S8**). Their expression patterns were similar to those of SiLRR-RLK3-5, illustrating that SiLRR-RLK8-6 may form a complex with SiLRR-RLK3-5 and coregulate plant immunity through the BR pathway.

SiLRR-RLK3-14 is homologs of NIK1 (NSP-interacting kinase 1), and the abundance of their transcripts was downregulated at early stage (**Supplementary Table S8**), which coincides with the fact that NIK1 acts as a negative regulator in plant immunity (Li et al., 2019). BRL3 (BR insensitive1-like 3) in *Arabidopsis* not only senses FLG22 and regulates ROS homeostasis (Tunc-Ozdemir and Jones, 2017) but also increases permeating agents such as proline in plants, which can improve plant drought tolerance without a penalty in growth (Fàbregas et al., 2018). In sesame, SiLRR-RLK8-7, as a homolog of BRL3, exhibited a upregulation trend during *M. phaseolina* infection, suggesting that SiLRR-RLK8-7 may play a crucial role in disease resistance (**Supplementary Table S8**).

The previous results of functional annotation, KEGG enrichment, promoter analysis, expression pattern and PPI network of SiLRR-RLKs all showed that SiLRR-RLKs are crucial in plant immunity. Thus, a coexpression network of SiLRR-RLKs under *M. phaseolina* stress was constructed. Interestingly, two coexpression immune modules were inferred in the core gene set based on PPI and coexpression network (**Figures 11**, **12**). They are the *SiLRR-RLK12-3/SiLRR-RLK12-13/SiLRR-RLK12-8/SiLRR-RLK9-6* module, which is homologous to the *Arabidopsis* BAK1/PEPR1/RLK7/SOBIR1/MIK2 complex, and *SiLRR-RLK10-8/SiLRR-RLK9-20* module, which is homologous to the *Arabidopsis* SRF8/PXC3/IRK complex. In *Arabidopsis*, the function of the SOBIR1/BAK1 complex has been well studied, and exogenous NLP20 treatment stimulates the formation of the BAK1/SOBIR1/RLP23 complex and initiates immunity (Gao et al., 2009). Furthermore, the SOBIR1/BAK1 complex could enhance the resistance of *Arabidopsis* to the fungi *P. infestans* and *S. sculrotiorum* (Liu et al., 2016). In addition to BAK1, the function of SOBIR1 in other species has also been studied in detail. In tomato, the homolog of SOBIR1 interacts directly with the disease resistance genes CF-4 and VE1, which mediated the resistance to the fungi *Cladosporium fulvum* and *Verticillium dahliae* (Liebrand et al., 2013). SOBIR1 in cotton was reported to interact with bHLH171, phosphorylate bHLH171 and confer resistance to the fungus *V. dahliae* (Zhou et al., 2019). In tobacco, SOBIR1 can fine-tune ROS production involved in the immune response to the fungus *Cladosporium fulvum* (Huang et al., 2021). These results suggest that the *SiLRR-RLK12-3/SiLRR-RLK12-13/SiLRR-RLK12-8/SiLRR-RLK9-6* module might be important in resistance to the fungus *M. phaseolina* (**Figures 11**, **12**). They might mediate resistance to the fungus *M. phaseolina* independently or form dimers or polymers with each other to mediate the immune response to *M. phaseolina* jointly, which needs further study. However, studies on the immune function of the SRF8/PXC3/IRK (Inflorescence and root apices receptor kinase) module are not clear. In *Arabidopsis*, PXC3 (Phloem intercalated with xylem-correlated 3) has been shown to interact with BAK1 to regulate vascular development (Xu et al., 2021), but whether it has a function in plant immunity is unknown. *Arabidopsis* SRF3 (Strubbelig receptor kinase 3) could coordinate immune responses, growth and development in plants (Platre et al., 2022), but the function of SRF8 is uncharted. Therefore, the role of the *SiLRR-RLK10-8/SiLRR-RLK9-20* module resistance to *M. phaseolina* in sesame is not clear, and further experiments are needed to solve this issue.

## 5 Conclusions

Whole genome identification and comprehensive analysis of the *SiLRR-RLK* gene family were carried out in this study. Phylogenetic, structural, evolutionary and expression profile analyses of *SiLRR-RLKs* revealed the complexity and diversity of the *LRR-RLK* gene family in sesame and its potential roles under *M. phaseolina* stress. Furthermore, we found several *SiLRR-RLK* genes that contributed to resistance to *M. phaseolina*. Altogether, the results provided a framework for further functional study of *SiLRR-RLK* genes.

## Data availability statement

The datasets presented in this study can be found in online repositories. The names of the repository/repositories and accession number(s) can be found in the article/**Supplementary Material**.

## Author contributions

WY: Data curation, Formal analysis, Investigation, Visualization, Writing – original draft. YN: Data curation, Resources, Writing – review & editing. HZ: Data curation, Resources, Writing – review & editing. XL: Data curation, Resources, Writing – review & editing. MJ: Data curation, Resources, Writing – review & editing. XZ: Resources, Writing – review & editing. YL: Resources, Writing – review & editing. HM: Conceptualization, Funding acquisition, Supervision, Writing – review & editing. HL: Conceptualization, Funding acquisition, Supervision, Writing – review & editing. HZ: Conceptualization, Funding acquisition, Supervision, Writing – review & editing.

## Supplementary material

Supplementary Figure 1 Statistics of *SiLRR-RLK* genes regulated by TFs (Genes with TF binding sites were considered to be regulated by TFs).

Supplementary Figure 2 Phylogenetic analysis and motif compositions of LRR-RLK proteins in six dicotyledons. Green hollow pentacles highlight SiLRR-RLK proteins.

Supplementary Figure 3 Expression profiles of *SiLRR-RLKs* during seed development (S1, S2, S3 and S4).

Supplementary Figure 4 Expression profiles of *SiLRR-RLKs* of Cluster 1 **(A)**, Cluster 2 **(B)**, Cluster 3 **(C)**, Cluster 4 **(D)**, Cluster 5 **(E)** and Cluster 6 **(F)** during *M. phaseolina* infection (0 - 48 h).
