# Supplementary figures and images for: Comprehensive analysis of sesame LRR-RLKs: structure, evolution and dynamic expression profiles under Macrophomina phaseolina stress
Source: Front Plant Sci. 2024 Feb 12;15:1334189. doi: 10.3389/fpls.2024.1334189 (PMC10895033; doi:10.3389/fpls.2024.1334189)

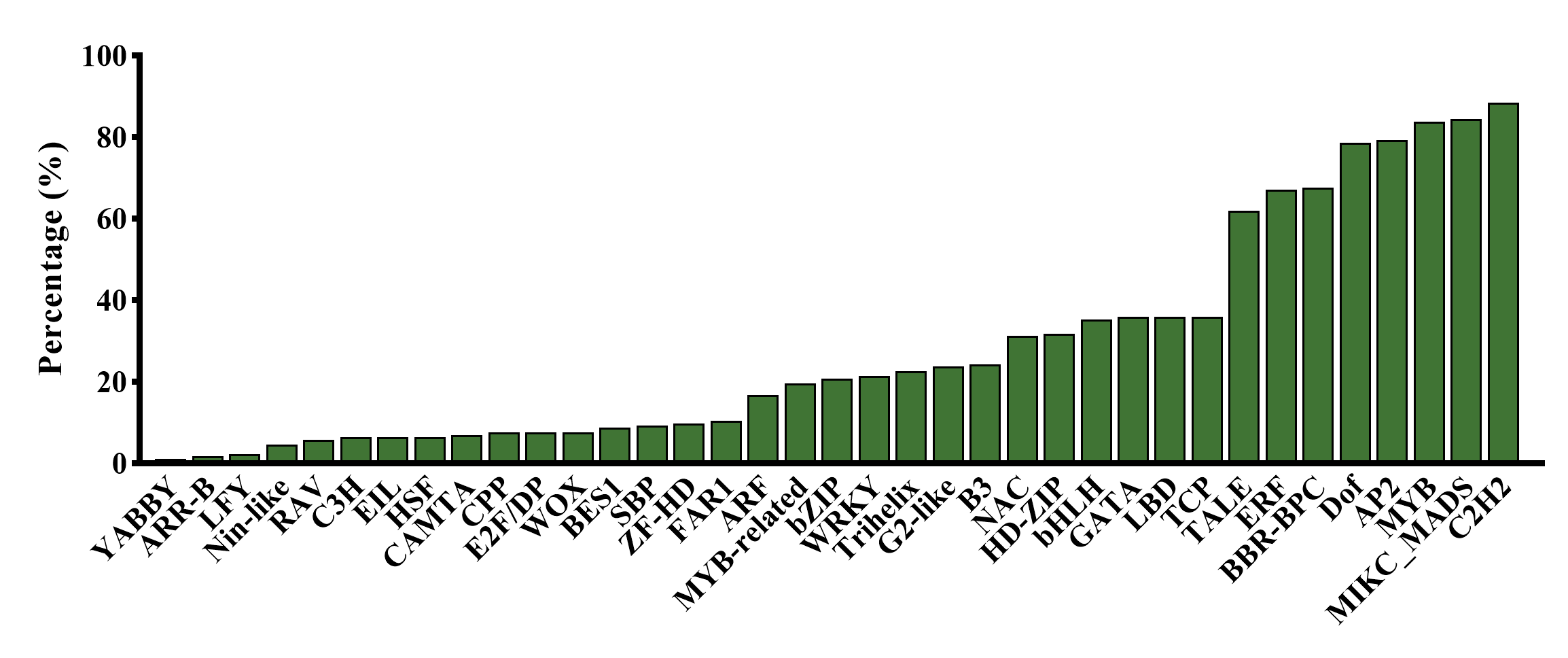

Supplement: Supplementary Figure 1 — Statistics of SiLRR-RLK genes regulated by TFs (Genes with TF binding sites were considered to be regulated by TFs). [file DataSheet_1.zip › Image 1 (67).TIF]

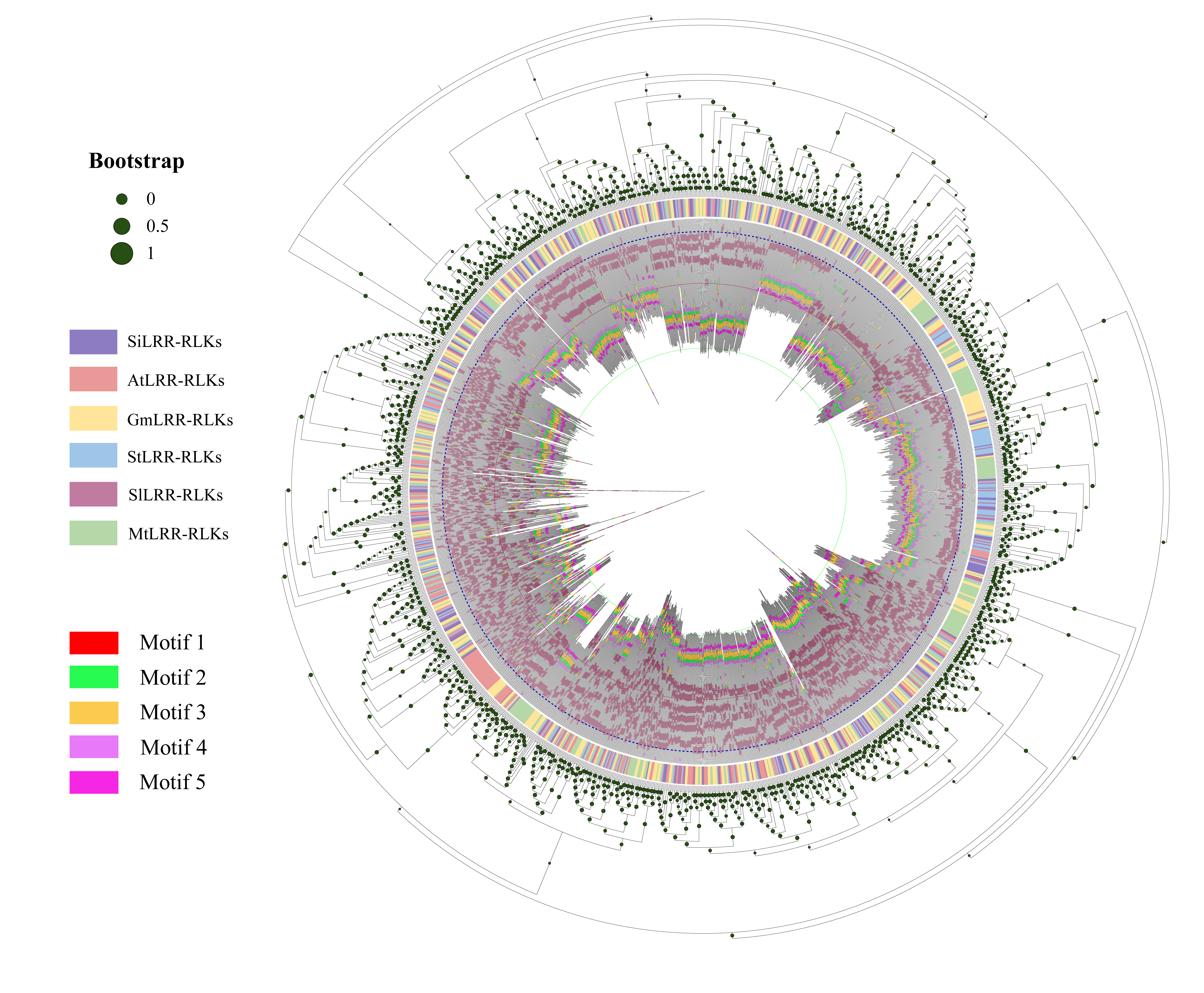

Supplement: Supplementary Figure 1 — Statistics of SiLRR-RLK genes regulated by TFs (Genes with TF binding sites were considered to be regulated by TFs). [file DataSheet_1.zip › Image 2 (40).TIF]

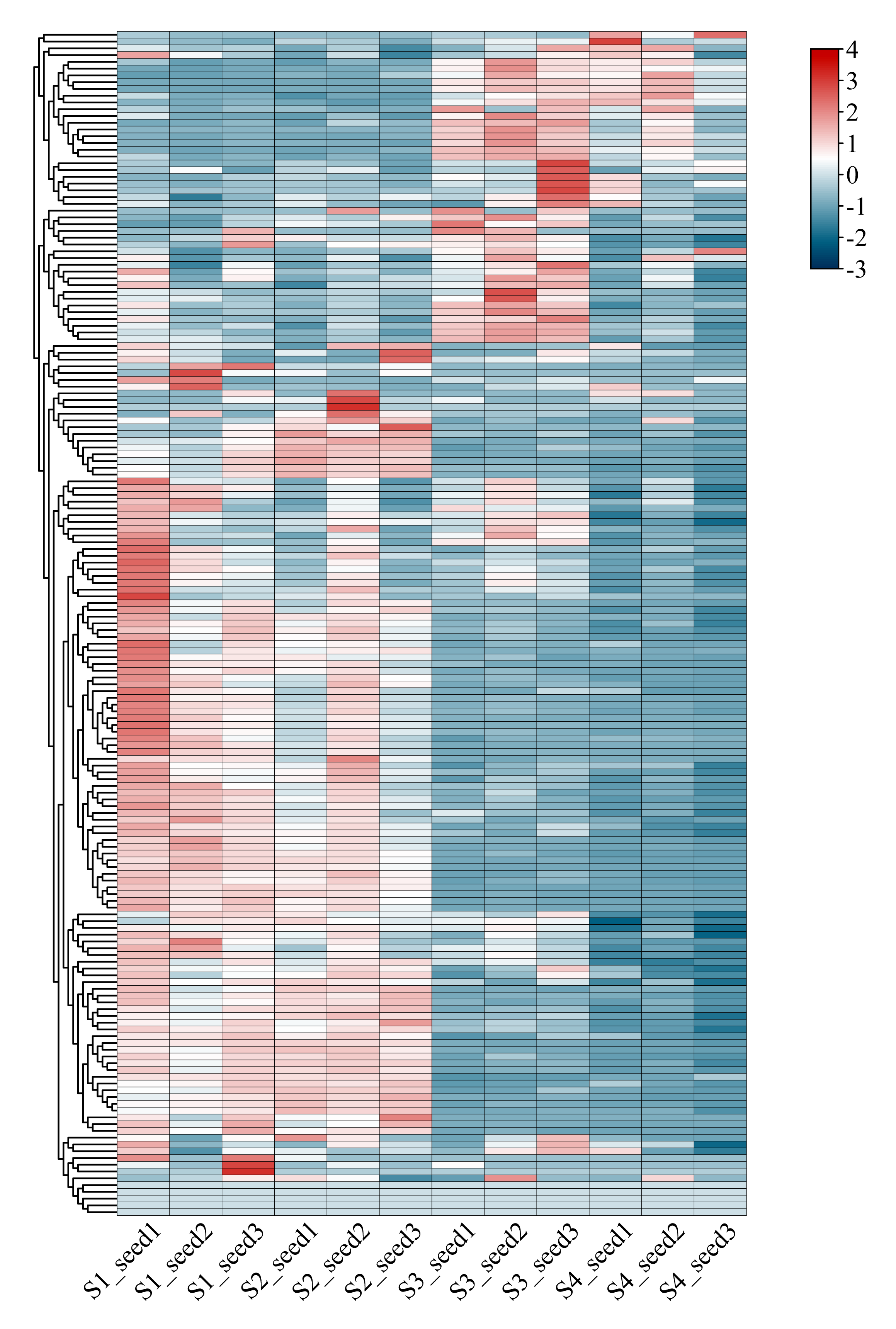

Supplement: Supplementary Figure 1 — Statistics of SiLRR-RLK genes regulated by TFs (Genes with TF binding sites were considered to be regulated by TFs). [file DataSheet_1.zip › Image 3 (23).TIF]

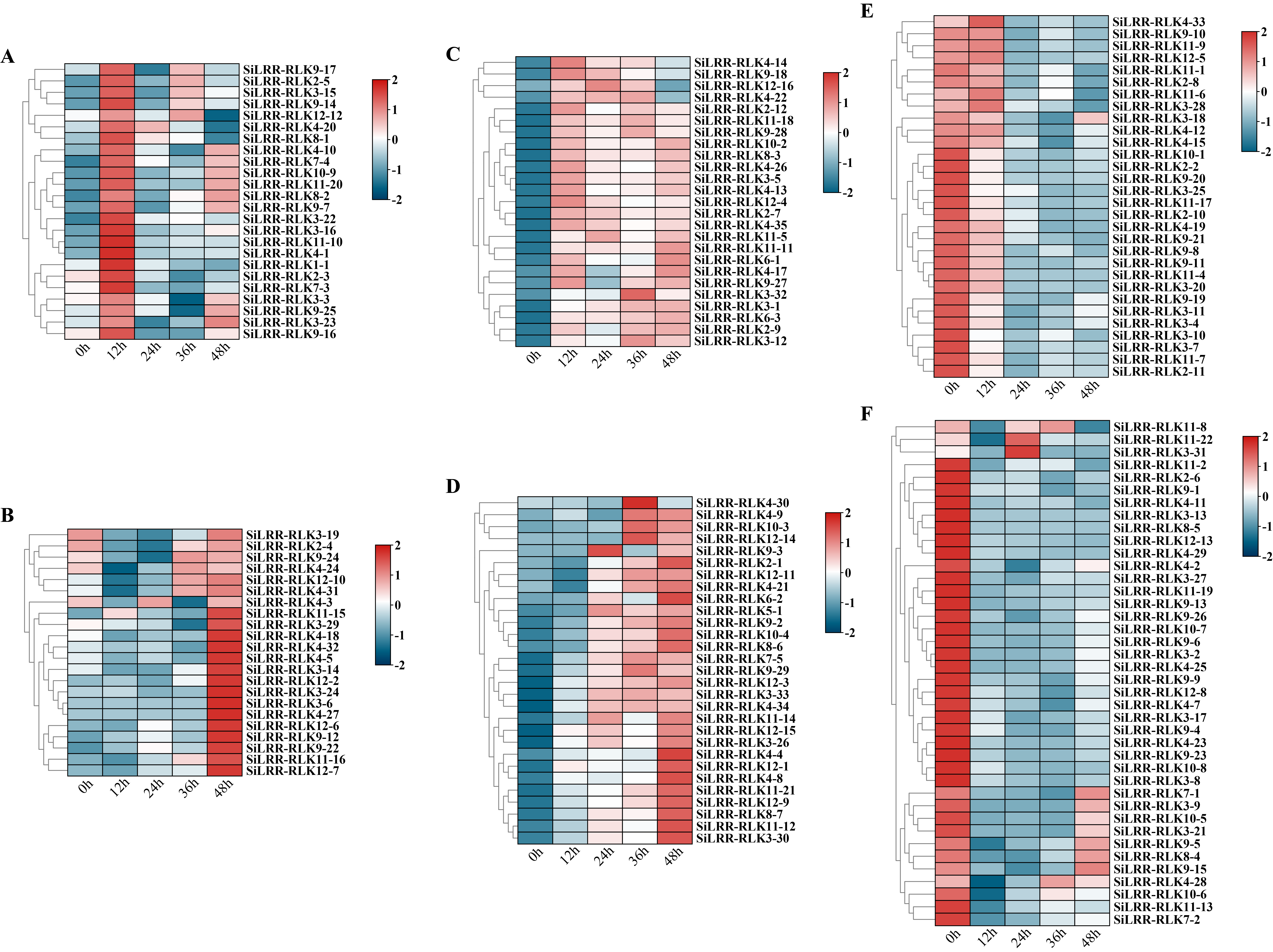

Supplement: Supplementary Figure 1 — Statistics of SiLRR-RLK genes regulated by TFs (Genes with TF binding sites were considered to be regulated by TFs). [file DataSheet_1.zip › Image 4 (18).TIF]
